# Association studies including genotype by environment interactions: prospects and limits

**DOI:** 10.1186/1471-2156-15-3

**Published:** 2014-01-06

**Authors:** Abdoul-Aziz Saïdou, Anne-Céline Thuillet, Marie Couderc, Cédric Mariac, Yves Vigouroux

**Affiliations:** 1 Institut de Recherche pour le Développement, UMR DIAPC IRD/INRA/Université de Montpellier II/ Montpellier SupAgro, BP64501, 34394 Montpellier, France; 2 Institut de Recherche pour le Développement, UMR DIAPC IRD/INRA/Université de Montpellier II/ Montpellier SupAgro, BP11416 Niamey, Niger; 3 Université Abdou Moumouni, BP 11040 Niamey, Niger; 4 Montpellier SupAgro, 2, place Pierre Viala, 34060 Montpellier, France; 5 Current address: UMR CEFE, 1919 Route de Mende, Montpellier, France; 6 Institut de Recherche pour le Développement, 911, avenue Agropolis, 34394 Montpellier, France

**Keywords:** Association study, G × E, Power simulation, Model selection, REML, PHYC, Vgt1

## Abstract

**Background:**

Association mapping studies offer great promise to identify polymorphisms associated with phenotypes and for understanding the genetic basis of quantitative trait variation. To date, almost all association mapping studies based on structured plant populations examined the main effects of genetic factors on the trait but did not deal with interactions between genetic factors and environment. In this paper, we propose a methodological prospect of mixed linear models to analyze genotype by environment interaction effects using association mapping designs. First, we simulated datasets to assess the power of linear mixed models to detect interaction effects. This simulation was based on two association panels composed of 90 inbreds (pearl millet) and 277 inbreds (maize).

**Results:**

Based on the simulation approach, we reported the impact of effect size, environmental variation, allele frequency, trait heritability, and sample size on the power to detect the main effects of genetic loci and diverse effect of interactions implying these loci. Interaction effects specified in the model included SNP by environment interaction, ancestry by environment interaction, SNP by ancestry interaction and three way interactions. The method was finally used on real datasets from field experiments conducted on the two considered panels. We showed two types of interactions effects contributing to genotype by environment interactions in maize: SNP by environment interaction and ancestry by environment interaction. This last interaction suggests differential response at the population level in function of the environment.

**Conclusions:**

Our results suggested the suitability of mixed models for the detection of diverse interaction effects. The need of samples larger than that commonly used in current plant association studies is strongly emphasized to ensure rigorous model selection and powerful interaction assessment. The use of ancestry interaction component brought valuable information complementary to other available approaches.

## Background

Deciphering the genetic basis of quantitative trait variation is a major challenge in biology. Linkage mapping and association mapping are two complementary methods that are widely used to study the relationship between genotype and phenotype. Linkage mapping or *family mapping*[1] is generally based on the progeny of experimental crosses. Association mapping (or population mapping) benefits from large populations which have inter-crossed for many generations, allowing a high number of recombination events to occur [1]. This historical recombination between loci generally leads to a very fine scale for genotype-phenotype association analysis [2].

One major pitfall of this method is that the genetic background of the populations could produce confounding effects which bias the statistical analysis and inflate the rate of false positives [3]. Methodological solutions have been developed to overcome this bias. First, methods were developed to analyse multi-locus molecular data from mapping samples to infer population structure [4-10] and to infer kinship relationships between individuals [11-13]. Second, satisfactory statistical models were proposed to correct for background effects using genetic relationship matrices [14-16].

Association mapping is especially powerful for common alleles and for moderate to large effects [2,17]. Association studies will certainly accelerate the study of genotype-phenotype relationships [18]. In particular, genome-wide association studies (GWAS) are promising ways to exhaustively identify polymorphisms linked to the traits of interest [1,18]. GWAS have already proved to be very useful in plants and have enabled the identification of a number of variants linked to phenotype [19,20].

The number of association studies performed in plants is currently increasing (see reviews [21-23]). However, most of these studies focused on the analysis of the main effect of molecular polymorphism on the phenotype (*i.e.* the effect of single factors with no interactions). To date, only a few association mapping studies reported tests of interaction effects (e.g. [24-28]). Interaction effects include genotype by environment interactions (G × E) and epistatic interactions between the genetic factors themselves. Genotype by environment interaction occurs when there is variation among genotypes in the rank order or relative magnitude of effects in different environments [29]. And, epistasis occurs when there is a statistical deviation from the additive combination of two loci in their effects on a phenotype [30,31].

In a study of *Drosophila melanogaster* populations, about 50% of phenotypic variation in adult olfactory behaviour was assigned to G × E, highlighting the importance of interactions in the architecture of this trait [32]. In maize Nested Association Mapping populations [33], the proportion of variance explained by G × E or epistatic interactions in flowering traits was low compared to genetic variance due to QTL main effects [34]. But studies in rice [35] and Arabidopsis [36,37], highlighted the considerable contribution of epistatic effects in determining flowering time for these species. Furthermore, exhaustive association studies revealed that, in general, QTLs explain only a part of trait heritability, even when a large number of loci are considered [34,38]. In human genetics, this gap is referred to as missing heritability [39]. G × E and epistatic interactions are among the effects that are expected to explain a fraction of this missing heritability [1,22,40].

So there is a strong interest for the extension of the plant association mapping framework to deal with interactions effects. The mixed linear model (MLM) framework [16] is the most commonly used in plant association mapping. A methodological examination of the MLM association mapping framework was undertaken to analyze epistatic interactions using population background [30,41]. A parameterization of mixed model variance structure was recently proposed to handle correlated traits [42]. This study examined pleiotropic effects and two way interactions between gene and environment. In our study, we proposed another strategy to include interaction component in mixed linear model. We proposed to consider interaction between SNP by environment (S × E) as well as to investigate structure by environment interaction (Q × E), SNP by structure interaction (Q × S) and three way interaction between SNP, ancestry and environment (Q × S × E). We think there is actual case where such interactions might be biologically useful and easy to explain. For example, Q × E deals with interaction between structure (represented by ancestry measure) and environment. Such interaction might be difficult to assess on a SNP basis if a trait is associated with numerous genes with small additive effects. However, the effect will be observed in this term Q × E if these numerous genes are associated with the structuration of populations (i.e. associated with adaptation). This term might be particularly useful to investigate for researcher working on ecological adaptation, because it addresses somewhat different phenotypic response in function of the population background (something close to plasticity at the scale of the population). Similarly, the Q × S might be an interaction of a major locus and several minor alleles fixed in the considered population (associated with population structure). These minors allele might not show up on a SNP by SNP analysis. Finally the three way interaction is a combination of the two interactions Q × S × E. Scenarios of interactions involving ancestry have not yet been examined in the context of plant association mapping. Also, previous studies used the variance structure of mixed model (random effects parameterization) [30,41,42]. The setting of interactions effects into the mean structure of mixed model (fixed effects) is a common practice. So the assessment of mixed model based on this alternative parameterization is also of interest but this has not been undertaken yet.

The aim of the present study was to investigate the use of a mixed linear model in an association mapping framework to identify genotype by environment interactions and interaction with population structure.

## Methods

### Linear mixed model specification

The commonly used mixed model in plant association mapping studies is:

y=Xβ+Qv+Sα+Zu+e,

where y is the vector of phenotype, β is a fixed effect other than SNP or population structure, α is the vector of a given SNP fixed effect, v is the vector of population structure fixed effects, u is the vector of polygenic background effects, and e is the residual error vector [16]. Q is the population ancestry matrix. X, S*,* Z were 0/1 incidence matrices relating y to β, α and u vectors respectively. The variance of the random effect u is expected to be Var(u) = K V, where K is the kinship matrix and V is the genetic variance. In the rest of this paper, we will use a simplified notation:

$$Y=\mu +E+Q+S+K+e\left(\mathrm{model}\;1\right)$$

Y is the phenotypic trait, μ is the intercept, E is a fixed effect other than population structure effect or SNP effect (e.g. an environmental effect), Q is the fixed effect of population structure, S is the fixed effect of a SNP (or any gene polymorphism). Q is set by matrices of population membership (e.g. ancestry matrices or principal components). K is the polygenic background random effect and e is the random residual of the model. K is set by a matrix of kinship relationship between individuals.

In this canonical form, the linear mixed model has mainly been used so far to measure the main effect of genetic polymorphism (and other covariates) on the phenotype. We first developed an extension of this model to fit gene by environment interaction (S × E). The term for S x E in the model is as follows:

$$Y=\mu +E+Q+S+S\;\times \;E+K+e\;\left(\mathrm{model}\;2\right)$$

If the environmental variable (E) is set as a random effect, the S × E term has to be set as random too. But, in this study, we consider the case where both S and E are fixed effects, so the interaction could be set as a fixed effect and will contribute to the mean structure. Note that this second model assumes the absence of interaction between population background and environment, so that no term was included for such interaction.

Next, we developed a full extension of the model to fit two and three way interactions between factors:

$$\begin{array}{l} Y=\mu +E+Q+S+Q\;\times \;E+S\;\times \;E+Q\;\times \;S\\ \;+Q\;\times \;S\;\times \;E+K+e\;\left(\mathrm{model}\;3\right) \end{array}$$

Q × E is the effect of interaction between ancestry and environment; Q × S is the interaction between SNP and ancestry; and Q × S × E is the three way interaction between ancestry, SNP and environment. All interactions are considered as fixed effects. Here, interaction between environment and genetic background is taken into account based on Q, and background effects linked to kinship are included only as random main effects.

### Basic scheme for the simulation of association mapping data

A basic model of genotype-phenotype association consists in simulating datasets characterized by the main effect of a single locus on the trait. Such an effect could be added on real phenotypic scores from a structured panel, so that the generated phenotype is linked to a real genetic background. With this scheme, there is no longer any need to simulate background matrices. So let us consider a panel of n individuals. We denote p_i_ the original (real) phenotype of an individual i. Considering a binary causative polymorphism (for instance presence/absence of a SNP), a simulated phenotype y_i_, can be assigned to each individual i as follows:

(1) $$y_{i}=p_{i}+S_{i}\;r\;\sigma_{G}+\epsilon_{i},$$

where S is a random variable with possible values “1” and “0”, standing respectively for the presence or the absence of the causative allele; σ_G_ is the standard deviation of the original trait across the whole panel; r is the genetic effect ratio (or *effect ratio*, i.e. a numeric variable that modulates the size of the effect as a function of σ_G_); and ϵ_i_ is a random variable that adds noise to the trait. The random variable S follows a Bernoulli distribution with P(S = 1) = q, q being the expected frequency of the causative allele. The random variable ϵ follows a normal distribution with a mean of 0 and a variance σ_ϵ_^2^. The variance σ_ϵ_^2^ is set to satisfy a given trait heritability h^2^ = σ_G_^2^/ (σ_G_^2^ + σ_ϵ_^2^). Note that the simulated SNP effect is independent of population structure and kinship relationship between individuals.

Hereafter, this basic simulation scheme was extended to generate data patterns with genotype by environment interactions and/or background interactions.

### Simulation of a gene by environment interaction

**Figure 1:**
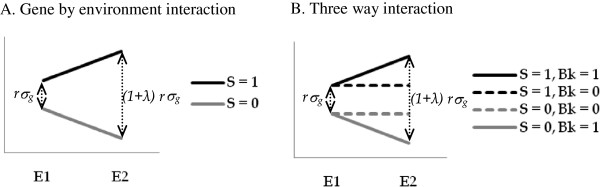
**Modeling of genotype by environment interactions.** Trait values (y axis) are presented in two environments, E1 and E2. **A)** The value of an individual phenotype is given with respect to the presence (1) or absence (0) of an SNP (S); rσg is the effect of the SNP in E1; (1 + λ) rσg is the effect of the SNP in E2. The coefficient λ is a numerical value that quantifies the change in the SNP effect from one environment to another. **B)** A marker correlated with population background is included, in addition to the S marker. The variable Bk denotes the presence (1) or absence (0) of this marker. The SNP effect (rσg) remained stable from environment E1 to E2 when Bk was absent. The size of this SNP effect varied with environment only in the presence of Bk.

A phenotypic variation caused by a single SNP was simulated in two virtual environments E_1_ and E_2_ (Figure 1A). The trait simulated in E_1_ follows the basic simulation scheme described in the previous paragraph. A new term was added in the simulation model for E_2_, to make the effect of the SNP vary between environments proportionally to a coefficient λ. This simulation model was specified as follows:

(2) $$\begin{array}{l} \mathrm{In}\;E_{1},y_{i}=p_{i}+S_{i}\;r\;\sigma_{G}+\epsilon_{i}\\ \mathrm{In}\;E_{2},y_{i}=p_{i}+S_{i}\;r\;\sigma_{G}+\left(S_{i}–q\right)\lambda \;r\;\sigma_{G}+\epsilon_{i} \end{array}$$

where λ is a numerical variable. The frequency of the causative allele (q) is taken into account to obtain an equal mean phenotype (y) between the two environments E_1_ and E_2_. In other words, this model simulates a null average main effect of environment, but specifically generates a gene by environment interaction.

The simulation was performed based on the two inbred panels described below (pearl millet and maize). Field experiment-based phenotypic scores (flowering time) from each panel were used to set the original phenotypic values p_i_. The effect ratio r varied across iterations from 0 to 1.5 (18 values); λ varied from 0.05 to 1 (4 values); q was set at 0.05, 0.25 and 0.5, respectively; and h^2^ was set to 0.25 and 0.75, respectively. The values of r and λ where chosen in the respectively defined ranges with regular spacing, to allow assessing the trends of power variation with respect to these parameters. The parameter σ_G_ was fixed at the value of the standard deviation of the original trait in each panel (σ_G_ = 6.83 days for pearl millet and σ_G_ = 8.72 days for maize). Population size was set at the value of original panels (n = 90 for pearl millet and n = 277 for maize), so that all available individuals were included. For each combination of parameters, the simulation was replicated a thousand times for pearl millet and a hundred times for maize. The lower number of iterations for maize allowed simulations to be run in a reasonable timescale. So in this step, around 12 million individual genotype-phenotype data points were simulated for maize and around 40 million for pearl millet. The simulation parameters are summarised in Table 1.

**Table 1:** Summary of parameters used in simulation

**Symbol**	**Definition**	**Range**
n	Sample size	90^a^ to 277^b^
p_i_	Original phenotype of individual i	Field-based flowering time score ^a,b^
r	Effect ratio, difference of effect associated with a variant	0 to 1.5
σ_G_	Standard deviation of the phenotype	6.83^a^; 8.72^b^
λ	Numeric value measuring the change in SNP effect between environments	0.05 to 2
q	Mean SNP allele frequency	0.05; 0.25; 0.50
q_0_	Mean ancestry	0.28^a^; 0.49^b^
h^2^	heritability	0.25; 0.75

^a^based on pearl millet real dataset.

^b^based on maize real dataset.

### Simulation of an interaction between population background, gene and environment

We considered two loci in a structured panel of n individuals: a randomly distributed SNP with a frequency expectation q and a background marker (Bk) linked to the ancestry in population P_0_. P_0_ was one of the populations in the panel. The SNP and the background marker were not linked. Two environments, E_1_ and E_2,_ were considered. We modelled the effect of the SNP on each individual phenotype so that this effect increased in the second environment only for individuals simultaneously carrying the causative allele at the SNP and the background marker Bk (Figure 1B). For each run, Bk was assigned to an individual if the ancestry coefficient of this individual in P_0_ (ranging from 0 to 1) was greater than a value randomly selected from a uniform distribution with the range 0-1. Given this approach, the expected frequency of the marker Bk in the sample was q_0_, the ancestry coefficient averaged from all individuals. At each iteration, the probability of an individual having the marker equals the ancestry of this individual in P_0_. The final phenotype was simulated using the following model:

(3) $$\begin{array}{l} \mathrm{In}\;E_{1},y_{i}=p_{i}+S_{i}\;r\;\sigma_{G}+\epsilon_{i}\\ \mathrm{In}\;E_{2},y_{i}=p_{i}+S_{i}\;r\;\sigma_{G}+\mathrm{Bk}\;\left(S_{i}–q\right)\;\lambda \;r\;\sigma_{G}+\epsilon_{i} \end{array}$$

This simulation scheme is therefore basically identical to the previous one (Equation 2), except for the presence of Bk which makes the effect variation depend on ancestry. In the model, Bk was set as a binary numerical variable scoring the presence or absence of the background-dependent marker (1 or 0, respectively). The parameter r varied from 0 to 1.5 (18 values) and λ varied from 0.05 to 2 (9 values); q was set at 0.05, 0.25 and 0.5, respectively; h^2^ was set at 0.25 and 0.75, respectively. For each combination of parameters, a thousand samples were generated for pearl millet panel and a hundred samples for maize, as in equation 2. The size of each individual sample was the same as in the original panel (n = 90 for pearl millet and n = 277 for maize). Among the populations comprising each panel, the population with the highest average ancestry (q_0_ = 0.28 for pearl millet and q_0_ = 0.49 for maize, respectively) was defined as P_0_, to maximize sample size. We wrote scripts with the software R (version 2.7.2) to implement all the simulation schemes used.

### Estimation of model components and assessment of power

The data simulating gene by environment interaction (Equation 2) were fitted using model 2. The model with two and three way interactions (model 3) was used to fit the data including the corresponding interactions (Equation 3). Model components were estimated using the REML method [43]. The fixed effects were significantly tested using the incremental Wald test procedure implemented in the ASReml package [43]. The power of each model was estimated for each parameter combination as the proportion of significant tests (P < 0.05) out of the total number of tests.

### The maize and the pearl millet panels

Results of independent field experiments were available for two panels (maize and pearl millet). Maize data came from Cornell University (Ithaca, New York). Pearl millet data came from the Institut de Recherche pour le Développement (IRD, France and Niger).

For pearl millet, the number of days from sowing to the female flowering stage was scored on a panel of 90 inbred lines. Nine field trials were carried out at different sowing dates throughout the rainy season, over a period of four years (2005 to 2008). For this dataset, we use the term *trial* to refer to the replicate of the field experiment at a given sowing date. All trials were conducted at the same location (Sadoré, Niger). Three of these trials (2005-2006) and the experimental design are described in detail in a previous paper [17]. The six additional trials (2007-2008) were conducted using the same experimental design, to study the trait over the whole sowing period in Niger. In 2007, two sowing dates were considered (June 16 and July 9), and four dates were considered in 2008 (June 26 and July 1, 7, 18). In each trial, approximately 7 to 10 plants per inbred line were measured for each trait to calculate the average phenotype for each inbred line. The averaged inbred flowering score in each trial was used in this study. The set of background markers for this panel (27 SSRs, 306 AFLPs) is described elsewhere [44,45]. Population structure was previously analyzed [16]. We computed a positive definite kinship matrix using the joint set of AFLP and SSR markers as previously described [17]. For candidate gene loci, we used seven SNPs with minor allele frequency q > 5% at the *PHYC* locus (sequence described in [17]).

The maize panel contained 277 inbred lines. Flowering phenotype (days to silk) was recorded in eight environments in the United States [34]. Hereafter, we use the term *trial* to refer to the maize experiment in a given year of experimentation at a given location. In this study, only seven trials were used, due to missing data in the eighth trial. For each inbred, the best linear unbiased predictor (BLUP) from each trial was used. Ancestry estimates were also available from a previous study which clustered maize inbreds into three populations: *NonStiffStalk* (NS), *StiffStalk* (SS) and *Tropical* (TS) populations [46]. Kinship coefficients were based on a set of previously described background markers [46] and calculated using the method implemented in the package EMMA [47]. The genotype score for a MITE insertion at the *Vgt1* locus was also available from a previous study [34]. One inbred was dropped from the sample due to missing data. The final sample size used in all parts of this study was thus 276.

### Statistical analyses

A common set of five competing models were defined to fit each of the maize and pearl millet datasets (Table 2). All five models were nested and differed from each other by one or more terms. For each panel, we compared the result of model selection using the Wald test (WLD) [48]. We also assessed the possibility to use information criteria (Additional file 1) and we finally used the Corrected Akaike Information Criterion (AICC) [49] and the Bayesian Information Criterion (BIC) [50]. AICC is an efficient information criterion and BIC is a consistent information criterion. For WLD, a larger model was considered better if at least one of the specific terms added in this model was significant (P < 0.05). To be more conservative for candidate gene association, we systematically set main effects and interactions involving environment or population background prior to effects involving candidate genes. Prior to statistical analyses, maize data were transformed using square root Box-Cox transformation (power of transformation 0.5) to minimize the departure of these data from model assumptions. A scaled variant of Box-Cox transformation adapted for mixed models [51] was used.

**Table 2:** Set of competing models compared for pearl millet and maize real data

**Model**	**Specification**	**Number of parameters**	
		**Maize**	**Pearl millet**
Fit1	Y = E + Q_i_ + S + (K + e)	12	18
Fit2	Y = E + Q_i_ + S + Q_i_ × E + (K + e)	24	66
Fit3	Y = E + Q_i_ + S + Q_i_ × E + S × E + (K + e)	30	74
Fit4	Y = E + Q_i_ + S + Q_i_ × E + S × E + Q_i_ × S + (K + e)	32	80
Fit5	Y = E + Q_i_ + S + Q_i_ × E + S × E + Q_i_ × S + Q_i_ × S × E + (K + e)	44	128

Y is the trait, E is environmental effect (trial effect), Q_i_ is population structure effect set by ancestry in the the i^th^ population (from k available populations, k-1 are used in the model), S is SNP effect. K is polygenic background random effect set by kinship matrix and e is the residual. A cross between terms represents interaction effect. For each dataset, the total number of model parameters is given.

## Results

### Power of the mixed model with a gene by environment interaction

**Figure 2:**
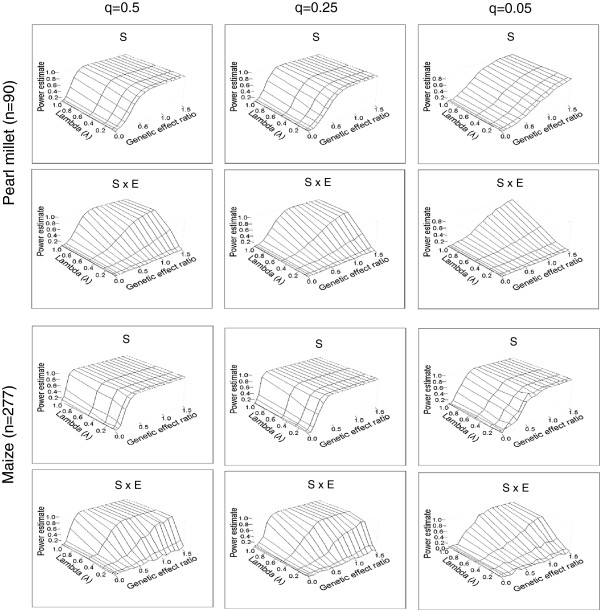
**Power of the mixed linear model to detect phenotypic effects including gene by environment interactions.** An SNP with differential effects across environments was simulated based on pearl millet and maize association mapping populations (see text for details). The main effect of the SNP (S) and the interaction between this SNP and the environment (S × E) were fitted with mixed linear model. The power of the model was calculated as the ratio of the number of runs in which a given effect was significantly detected out of the total number of runs. Power is plotted for h^2^ = 0.75 and according to allele frequency (q), genetic effect ratio (r) and λ. The parameter λ measured the variation in the magnitude of the SNP effect and the environment. The largest panel (maize, n = 277 individuals) performed globally better. However, the relative variation in power as a function of the parameters showed similar features in both panels. Power increased with an increase in r and was higher with common allele frequencies (q = 0.5, q = 0.25). The ability to detect the interaction was particularly sensitive to λ. The highest range of power (for example power > 80%) corresponded overall to relatively large parameter values. This indicates that these current mapping frameworks might be limiting for traits that are fundamentally shaped by loci with very small effects.

We used the simulated data to measure the power of mixed linear model to detect gene main effect and gene by environment interaction (Figure 2, Additional file 1: Figure S1). The power increased with the genetic effect ratio r and with the allele frequency in both maize and pearl millet samples. The power was highest with allele frequencies of 0.5 and 0.25 and with high heritability (h^2^ = 0.75). Also, the power to detect the interaction was more sensitive to the coefficient λ; this was expected because λ measures the increase in the effect of the SNP from the first environment to the second. The power was very low for the detection of gene by environment interactions when low heritability was combined with low allele frequency and/or low effect size. The global pattern of power variation with the parameters (r, λ, q) was consistent for both datasets (maize and pearl millet), but the maize sample performed globally better than the pearl millet sample in terms of absolute power. So in the maize sample, environmental interactions underlined by common alleles with relatively strong effects were still detectable at h^2^ = 0.25 and also the detection of rare alleles (q = 0.05) was better than that in pearl millet (Additional file 1: Figure S1). Finally, we calculated the simulated effect size resulting from the combination of parameters (Additional file 2: Table S1). For instance, SNP by environment interaction effects of 3.05 days and 4.44 days were detected for the flowering trait with a power of about 95% in both maize and pearl millet (for h^2^ = 0.75 and q = 0.5). The precision of estimation of fixed effects by this model was generally good with respect to the actually expected effects (R^2^ > 0.99; data not shown).

### Power to detect two and three order interactions

**Figure 3:**
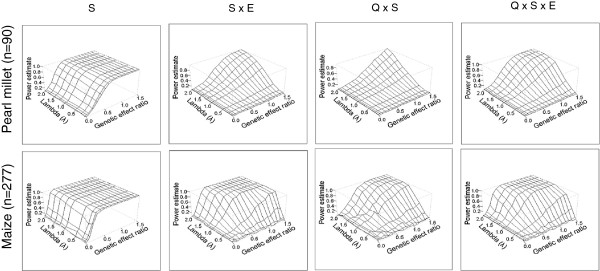
**Power of the mixed linear model to detect phenotypic effects including three way interactions.** The data were simulated using pearl millet and maize panels (see text for details). The set of simulated effects consists of SNP main effect (S), SNP by environment interaction (S × E), ancestry by SNP interaction (Q × S) and three way interaction between ancestry, SNP and environment (Q × S × E). The power to detect each effect is plotted for heritability h^2^ = 0.75 and the allele frequency q = 0.5, according to the effect ratio r, and the parameter λ which influences variations in the effect with the environment. Power increased with an increase in r for all effects, and a strong effect of λ was observed for the interactions. The highest range of power (say power > 80%) was reached only with relatively large effect size. Otherwise, there was a relative improvement in power for the maize panel (3 times larger than pearl millet panel).

We measured the power of MLM to detect two and three way interactions between SNP (S), ancestry (Q) and/or environment (E) (Figure 3, Additional file 1: Figures S2 and S3). The impact of the parameters (r, q, h^2^) on the power was globally similar to the impact described in the case of the gene by environment interaction. However, here, the power to detect the interactions appeared to be more sensitive to the value of differential effect between environments (λ). Thereby, even in the best allele frequency and heritability conditions (Figure 3), the three way interaction (i.e. Q × S × E) was not detected unless a critical value of λ was reached (roughly λ >1 in the present samples, in other words the main effect and the interaction effect should be of the same strength). A greater decrease in power was observed with h^2^ (Additional file 1: Figures S2 and S3). With h^2^ = 0.75 and q = 0.5, three way interaction effects of about 3.5 days or more were detected with a high power (>95%) in both panels (Additional file 2: Table S2). Note that results concerning complex interactions were not displayed at the lower allele frequency 0.05 in the pearl millet sample. Some ASReml-R runs aborted in this case due to the low number of individuals carrying the SNP and the background marker. The precision of estimation of fixed effects by this model was generally good with respect to the actually expected effects (R^2^ > 0.99; data not shown).

### Analysis of *PHYC* effect in the pearl millet association panel

We used the extended mixed linear models to analyze associations with the pearl millet flowering trait scored in a design including nine trials. We compared a set of five competing models for these data (Table 2). Model selection based on WLD (Additional file 2: Table S3) suggested a simple gene effect for all SNPs but the SNP in position 155. For this SNP, the best model selected by WLD included an interaction between *PHYC* and the environment, and this interaction effect was slightly significant (P = 0.0472). For the other SNPs, the main effect was significant while none of interaction effects were significant (Table 3). Using other criteria (AICC and BIC) illustrated the tendency already observed on the simulated data (Additional file 3 and Additional file 1: Figure S6). AICC systematically selected the full model with three way interaction and BIC systematically choose the simplest model without any interaction (Additional file 1: Figure S4).

**Table 3:** Effects significance for pearl millet data

**Effect**	**df**	**Fit1**	**Fit5**
Intercept	1	**< 10**^**-26**^	**< 10**^**-26**^
Trial	8	**< 10**^**-26**^	**< 10**^**-26**^
Q_1_	1	0.1954	0.1917
Q_2_	1	**0.0017**	**0.0016**
Q_3_	1	0.1663	0.1610
Q_4_	1	0.0244	0.0231
Q_5_	1	0.1128	0.1103
Q_6_	1	0.0519	0.0511
*PHYC*	1	**0.0044**	**0.0044**
Trial × Q_1_	8	-	0.2942
Trial × Q_2_	8	-	0.3272
Trial × Q_3_	8	-	0.1102
Trial × Q_4_	8	-	0.4017
Trial × Q_5_	8	-	0.6050
Trial × Q_6_	8	-	0.8051
Trial × *PHYC*	8	-	0.0658
Q_1_ × *PHYC*	1	-	0.5669
Q_2_ × *PHYC*	1	-	0.2556
Q_3_ × *PHYC*	1	-	0.1405
Q_4_ × *PHYC*	1	-	0.6343
Q_5_ × *PHYC*	1	-	0.6349
Q_6_ × *PHYC*	1	-	0.5684
Trial × Q_1_ × *PHYC*	8	-	0.1705
Trial × Q_2_ × *PHYC*	8	-	0.3126
Trial x Q_3_ × *PHYC*	8	-	0.8426
Trial × Q_4_ × *PHYC*	8	-	0.0537
Trial × Q_5_ × *PHYC*	8	-	0.0821
Trial × Q_6_ × *PHYC*	8	-	0.6632

The SNP considered here for *PHYC* is the SNP in position 101. The model selected by both BIC and WLD (Fit1) and, respectively, the model selected by AICC (Fit5) were considered. Fixed effects significance was assessed using Wald test. P-values are given for each fixed effect term in the model. Significant p-values (P < 0.05) are highlighted in bold. Q_i_: ancestry in population i; df: degree of freedom.

### Analysis of interactions in the maize association panel

The preliminary analysis of maize data using the standard framework (MLM with no interaction effects) produced different results depending on the trial (Additional file 2: Table S4, Additional file 1: Figure S5). The effect of *Vgt1* was significant in some environments (P < 0.05), but not in others (Additional file 2: Table S4, Additional file 1: Figure S5). However, the combined p-value obtained from these seven independent trials using Fisher’s method (Additional file 2: Table S4) supported a globally significant effect of *Vgt1* (P = 7.52 × 10^-5^). We set mixed linear models to test for possible interactions (Table 2). We performed a comparative model selection using WLD (Additional file 2: Table S3), AICC and BIC (Additional file 1: Figure S4). These criteria were expected to have a good performance of model choice given the current maize panel size (Additional file 3 and Additional file 1: Figure S7). The Wald test approach and AICC choose the model including a gene by environment interaction and an ancestry by environment interaction (Fit3). BIC choose a closely related interaction model in which the gene by environment interaction was dropped (Fit2).

The Wald test (Table 4) showed a significant effect of interaction between environment (trial location) and maize ancestry (P = 2.52 × 10^-10^ for the NS population, and P = 5.06 × 10^-18^ for the TS population). A highly significant environment main effect was also highlighted (P < 10^-26^), as expected with contrasting environments. Note that the main effect term set for *Vgt1* was not significant (P = 0.37). Instead, we observed a significant effect of interaction between this locus (*Vgt1*) and environment (P = 2 × 10^-6^, Table 4).

**Table 4:** Effects significance for maize data

**Effect**	**df**	**Fit2**	**Fit3**
Intercept	1	**< 10**^**-26**^	**< 10**^**-26**^
Trial	6	**< 10**^**-26**^	**< 10**^**-26**^
Q_NS_	1	**5.86 × 10**^**-05**^	**6.27 × 10**^**-05**^
Q_TS_	1	**4.21 × 10**^**-04**^	**4.43 × 10**^**-04**^
Vgt1	1	**0.373**	**0.379**
Trial × Q_NS_	6	**4.16 × 10**^**-10**^	**2.52 × 10**^**-10**^
Trial × Q_TS_	6	**1.25 × 10**^**-17**^	**5.06 × 10**^**-18**^
Trial × Vgt1	6	-	**2.00 × 10**^**-06**^

The model selected by BIC (Fit 2) and, respectively, the model selected by both AICC and WLD (Fit3) are considered. P-value of fixed effects (Wald test) is given for maize flowering time with respect to the fitted model. Significant p-values (P < 0.05) are highlighted in bold. Q_i_: ancestry in population i (see text); df: degree of freedom.

## Discussion

### Impact of data parameters on the power of mixed linear models

Previous association mapping studies in plants investigated the power of mixed linear model mainly in the simple case of main effects [10,13,16,17]. More recently, an assessment of the power of mixed model was reported for some cases of interactions in association mapping [42]. The concerned study highlighted the best efficiency of mixed model for epistasis and interaction detection, compared to other methods [42]. But the reported simulation did not make explicit assessment of the impact of genetic parameters as allele frequency and heritability on the power of the mixed model itself. For instance, only one arbitrary value of heritability was examined in this study for the *major locus scenario* (h^2^ = 0.95) and, respectively, for the *oligogenic scenario* (h^2^ = 0.5) [42], making it impossible to appreciate the loss of power due to trait heritability in either scenarios. Also, this simulation did not have explicit control on the effect of allele frequency and effect size.

In the current study, we simulated data based on an explicit control on heritability, allele frequency and effect size, to investigate how these parameters impact the power of mixed model. Diverse patterns of G × E interactions occur in biological data [52]. Our first model of interaction (Equation 2) was relatively simple and provided a learning case of a gene by environment interaction. The last presented scheme (Equation 3) was more complex and allowed the examination of higher order interactions.

While mixed model ensures an improved power for interaction detection compared to different methods [42], our study considered how this power inflates with respect to data parameters. Particularly, our results highlighted the drastic loss of power in association mapping due to low trait heritability and/or low allele frequency, whatever the type of effects considered (main effects and interaction effects). This suggest that the current association mapping framework will perform well only for SNPs with relatively high frequency (roughly q > 0.05) and for traits with relatively high heritability (h^2^ > 0.25). The framework will lack the identification of rare alleles or alleles affecting poorly heritable traits. The loss of power was more critical when higher order interactions were considered. One obvious statistical reason is that the power to detect higher order interaction (Q × S × E, for example) relies not on the allele frequency alone, but rather on the combination of the frequency of all variables involved in the interaction. As allele frequency is a proportion (i.e. a numeric value between 0 and 1), this combination is necessarily lower than any of the original variable frequencies. Furthermore, these simulations suggest that the exhaustive study of trait architecture based on current plant association panels will suffer from the bias of discarding not only rare alleles but also alleles with very small effects, because the full level of power (>0.95) could be reached only with less realistic effect sizes. For instance, the effect size needed to reach 95% of the simulated interactions in the best cases (h^2^ = 0.75 and q = 0.5) corresponded approximately to about 0.4 or 0.5 fold the standard deviation of the trait (Additional file 2: Tables S1 and Table S2).

Finally, we observed that the absolute level of power was improved in the simulations based on maize data compared to pearl millet. Although other properties of these two panels differed (e.g. population structure, trait variance), the improvement in power was in great part due to the effect of the larger sample size in maize (three times larger than the pearl millet sample). But despite this difference in the absolute value of power estimates, it is important to note that the pattern of power variation, as a function of allele frequency, heritability or effect size, was consistent for the two panels. To explore more parameters spaces in terms of genetic background configuration, allele frequency or sample size, the use of simulated panels based on theoretically defined parameters (rather than real panels) will be more suited. But this is not implemented in the current study.

Statistical limits to the analysis of complex interactions using small panels were revealed in when we used very low allele frequency for the smaller dataset (pearl millet data). The number of inbred baring the allele is then too low and the reliability of model components defined on the basis of rare combinations is statistically questionable. Taking all the simulation results together, it appears that no single parameter determines the power of the model and that the effects of all parameters might balance each other. For instance, a good allele frequency does not necessarily imply good power when heritability is insufficient, and reciprocally. In this connection, the power of the MLM framework for a particular set of data needs to be discussed with respect to the combination of data characteristics, instead of focusing on only one or two parameters. This will have implications for the design of association studies, in particular when interactions are of interest.

They are some parameters which could be more easily handled when planning a plant association study. For instance, the size of a panel can be extended with an appropriate sampling effort to reach a sample size favourable for the detection of effects. As revealed by the comparison of the pearl millet panel (n = 90) and maize panel (n = 277), considering a size of hundreds of individuals appear to be the minimum required to ensure a powerful design. However, even in this case, very small phenotypic effects or very low allele frequency also limit the performance of the MLM framework as already noted. To be able to detect rare alleles, the use of family mapping (linkage analysis) could be recommended [1]. Also, the use of original approaches combining linkage and association mapping as developed in maize proved very efficient [33,34].

Finally, the use of the present framework will be more suited for: 1) association mapping sample of several hundred individuals, 2) traits with high heritability, and 3) relatively common allele.

### Confirmation of *PHYC* effect into a multitrial mixed model framework

Methods of model selection led to contrasting results for pearl millet (Additional file 1: Figure S4). AICC selected the full model, whereas BIC and WLD selected the simplest model. Given the high difference in the number of parameters between the two selected models, it is unlikely that model uncertainty could conciliate these results, on the contrary of maize results (see below). This contrast is the result of statistical bias in model selection and would be mainly associated to the small size of pearl millet sample. This issue was addressed and examined in this study using a full simulation approach (Additional file 3). In the light of this simulation, we know that unlike for maize (n = 277), the current sample size in pearl millet (n = 90) does not allows a confident use of information criteria. Indeed, the simulation showed the occurrence of contrasting behaviour between methods of model selection when sample size is low. In particular, efficient criteria like AIC and AICC showed a bias toward over-parameterization, while consistent criteria like BIC and CAIC seemed more conservative (Additional file 3). It is also to be noted that, unlike information criteria, the standard procedure of model selection based on Wald test implies multiple testing and raises the problem of type I error control. For example, on pearl millet data, the number of tests needed to compare the 5 defined models was 19 tests per SNP, while the model selection based on BIC needs only 5 comparisons (Additional file 2: Table S3).

We used Wald test to assess the fixed effects considering respectively the full model and the simplest model (Table 3). Whatever the model considered, we confirmed the effect of *PHYC* polymorphism on the phenotype, and this confirmed a previous study based on the classical MLM framework [16]. This effect is set as main effect and we found no strong evidence of interaction with environment. The slight signal of gene by environment interaction detected with *PHYC* SNP in position 155 (P = 0.0472, Additional file 2: Table S3) is significant at the nominal threshold, but could not be statistically validated when we correct for multiple testing (for example, Bonferroni threshold would be about 0.005 for a correction for 9 tests). However, the small sample size could possibly explain the absence of significant test even in the presence of real interaction effect, due to lack of power.

### Statistical evidence in maize for two types of interactions

The result of model selection in maize data was very close between methods. This good consistency between the methods for this relatively large sample (n = 277) is actually expected in the light of our simulation study (Additional file 3). Two types of biologically important interactions are highlighted by these results. First, we found significant interactions between maize ancestry and environment. This suggests that differences in the background of the three maize populations led to a differential reaction to environment variation. The effect of the interaction between population structure and the environment suggests that these populations respond differently to different environments. The analysis population structure, we actually analyzed an unknown number of loci co-varying with the population structure. These loci will be difficult to identify on a SNP by SNP effect because their effect is absorbed by the structure effect of the mixed model (they co-varied with the structure). However, we showed here that there is interesting genetic feature at the population level that could be retrieved. These interactions could be interpreted as a form of plasticity at the scale of the population.

The second type of interaction was an interaction between one locus (*Vgt1*) and the environment, with a highly significant probability (P < 10^-6^). When we performed site by site analysis, this effect was also detected. The effect of the MITE insertion at this locus was in the same direction across all the environments examined, but the size and the significance of this effect was different between trial locations. The two types of interaction effects have important implications for the understanding of quantitative trait variation. More generally, interaction between biological and environmental factors impact trait stability, trait prediction, and trait architecture [53], which underlies the interest of these studies.

The present study is one of the first in plant to deal with genotype by environment interaction in a association mapping framework. It highlight the challenging issue about interaction study based on deep examination of important aspect of this issue (modelling of different types of interaction, model setting and model selection, model power, impact of data parameters like sample size and allele frequency, *etc.*). Nonetheless, other issues remained to be addressed. For example, it will be interesting to evaluate possible heterogeneous variance structure using mixed model framework.

## Conclusion

We used mixed linear model to discuss the statistical perspective of using association mapping panels to study interaction effects in plants. First, this study highlighted the need of large sample size to ensure best model selection and high power. Most of the association panels of plants species to date are composed of a fairly small number of individuals, so larger panels are to be recommended in the future to deal better with G × E interactions. Second, we showed a strong impact of data parameters on the power, with a drastic loss of power when allele frequency, heritability, or effect size, are low. This shortcoming reinforces the need of complementary methods to deal with rare alleles or alleles with small effects. However, we illustrated cases of success of the current mixed model framework by identifying, in the maize association mapping panel, two types of biologically interesting effects of interaction between genetic factors and environment. So the development of interaction studies based on this mixed model framework would contribute understanding quantitative trait variation, notably by taking into account key components of G × E interactions. This framework is complementary to the frameworks of association mapping developed to deal with multi-QTL [54,55], and QTL by QTL interactions [27,28].

## Competing interests

The authors declare no competing interest.

## Authors’ contributions

MC and CM sequenced pearl millet panel. CM, AAS, and YV designed and supervised field trials for pearl millet. AAS participated in the design of the study and performed statistical analyses. AAS, ACT, and YV wrote the paper. YV coordinated and supervised the study. All authors read and approved the final manuscript.

## Supplementary Material

Additional file 1 **This file provides additional figures cited in the text. Figure S1** Power of mixed linear model to detect phenotypic effects including gene by environment interaction. **Figure S2** Power of mixed linear model to detect phenotypic effects including 3-way interactions in the pearl millet sample. **Figure S3** Power of mixed linear model to detect phenotypic effects including 3-way interactions in the maize sample. **Figure S4** Model selection for the analysis of associations in a multi-trial design. **Figure S5** Effect estimates of the Vgt1 locus on days to silk for each of the 7 environments. **Figure S6** Model selection based on different criteria. **Figure S7** Frequency of selection of competing models as a function of sample size in maize. Click here for file

Additional file 2: Table S1 Minimum effect detected with a power of 95% in the model with gene by environment interaction. **Table S2** Expectation of minimum effect detected with a power of 95% in the model with three way interaction. **Table S3** Model selection based on WLD. **Table S4** Wald test for Vgt1 effect analyzed independently into each trial. **Table S5** Formulation of information criteria. **Table S6** Rate of selection of competing models by information criteria, at different values of effect size parameter r. **Table S7** Significance of the effect of data parameters on the rate of success of information criteria. Click here for file

Additional file 3 Assessment of methods for model selection. Click here for file
