# Supplementary material for: Association studies including genotype by environment interactions: prospects and limits
Source: BMC Genet. 2014 Jan 6;15:3. doi: 10.1186/1471-2156-15-3 (PMC3901036; doi:10.1186/1471-2156-15-3)
Supplement: Additional file 1 — This file provides additional figures cited in the text. Figure S1 Power of mixed linear model to detect phenotypic effects including gene by environment interaction. Figure S2 Power of mixed linear model to detect phenotypic effects including 3-way interactions in the pearl millet sample. Figure S3 Power of mixed linear model to detect phenotypic effects including 3-way interactions in the maize sample. Figure S4 Model selection for the analysis of associations in a multi-trial design. Figure S5 Effect estimates of the Vgt1 locus on days to silk for each of the 7 environments. Figure S6 Model selection based on different criteria. Figure S7 Frequency of selection of competing models as a function of sample size in maize. [file 1471-2156-15-3-S1.docx]

**Saïdou *et al.***

**Supplementary figures**

**Figure S1** Power of mixed linear model to detect phenotypic effects including gene by environment interaction.

**Figure S2** Power of mixed linear model to detect phenotypic effects including 3-way interactions in the pearl millet sample.

**Figure S3** Power of mixed linear model to detect phenotypic effects including 3-way interactions in the maize sample.

**Figure S4** Model selection for the analysis of associations in a multi-trial design.

**Figure S5** Effect estimates of the *Vgt1* locus on days to silk for each of the 7 environments.

**Figure S6** Model selection based on different criteria.

**Figure S7** Frequency of selection of competing models as a function of sample size in maize.


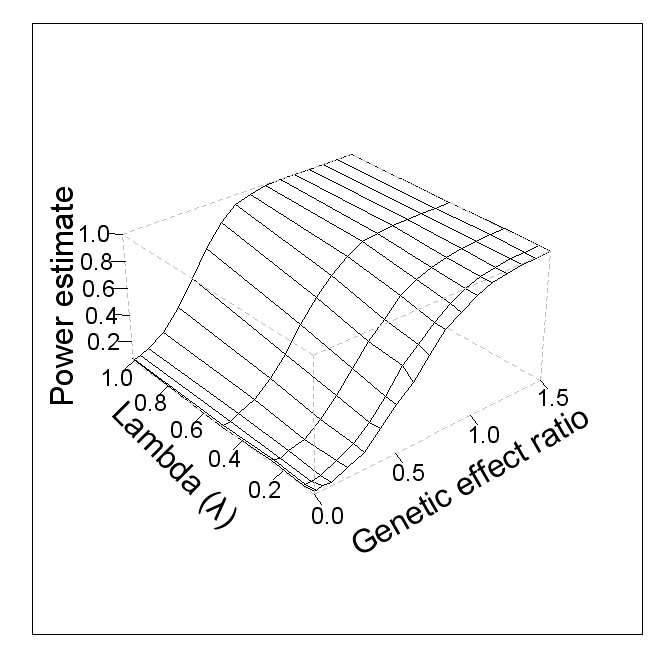


S


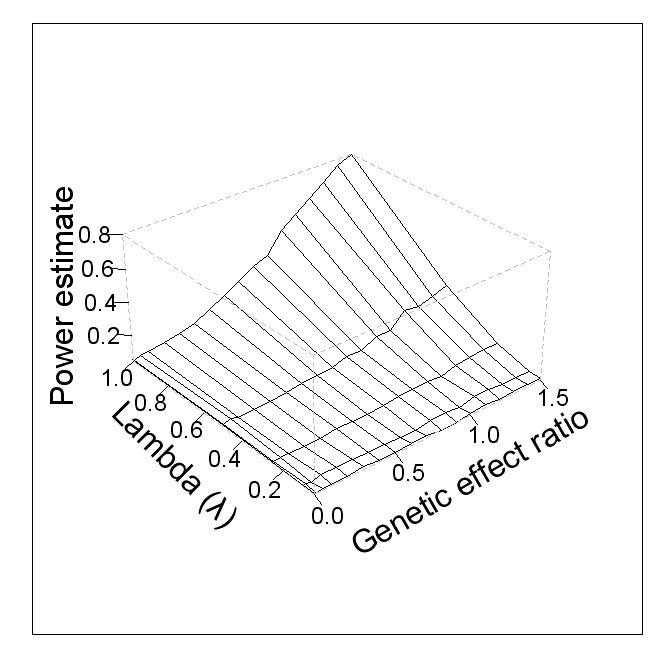


S x E

A. h^2^=0.25; q=0.5


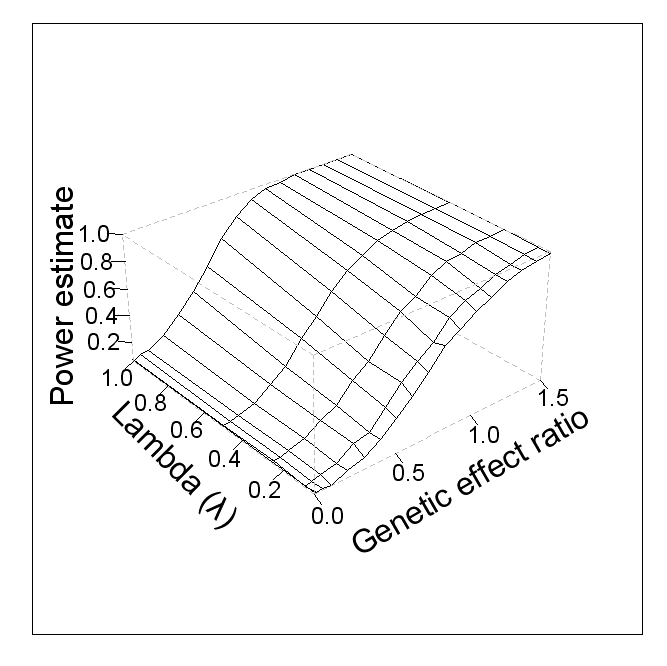


S


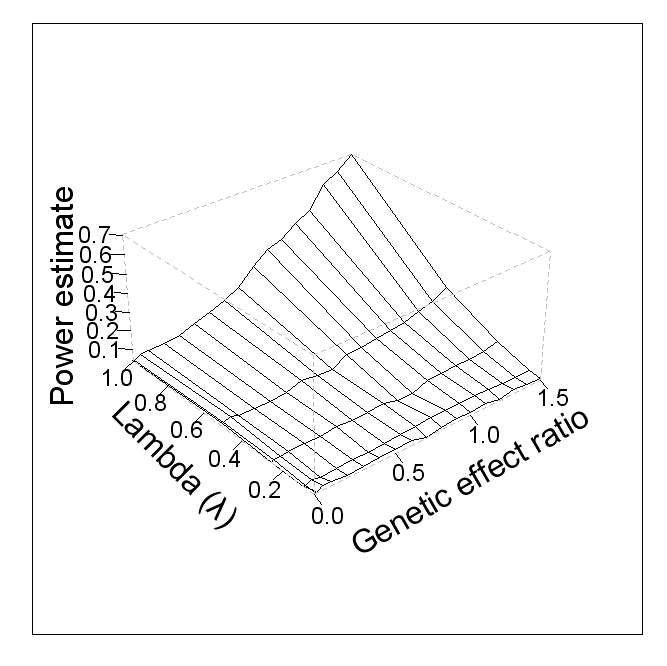


S x E

B. h^2^=0.25; q=0.25


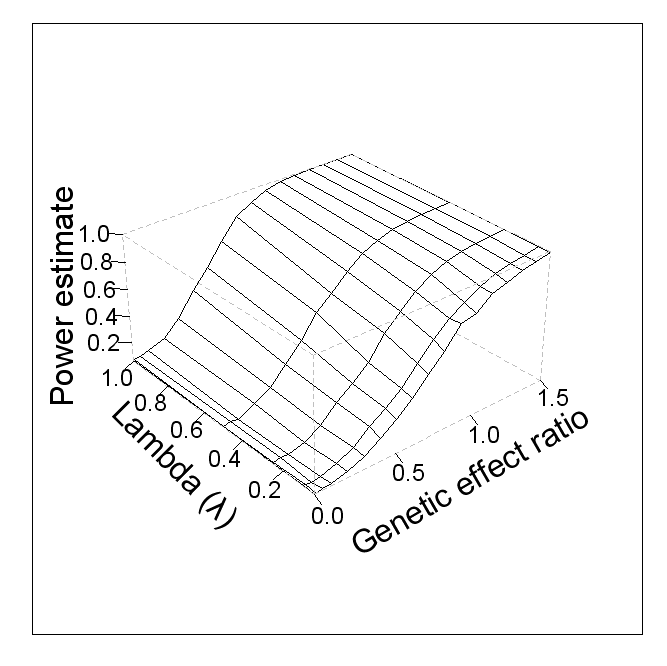


S


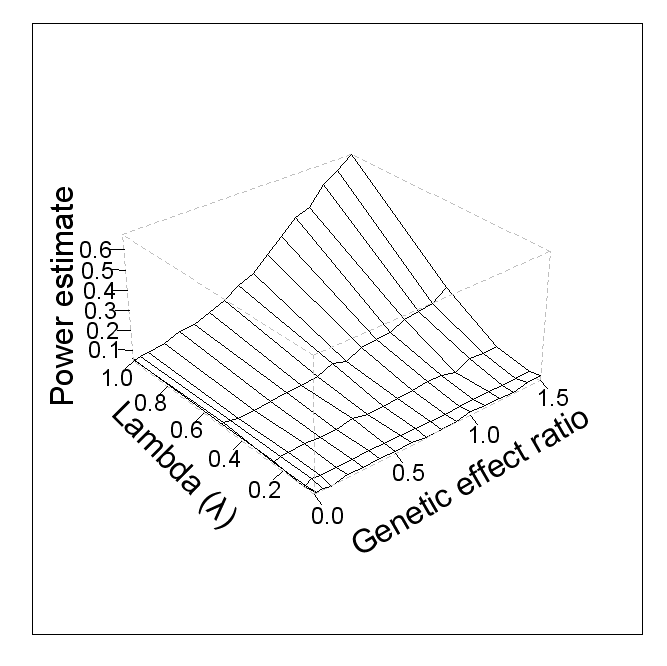


S x E

C. h^2^=0.25; q=0.05

D. h^2^=0.25; q=0.5


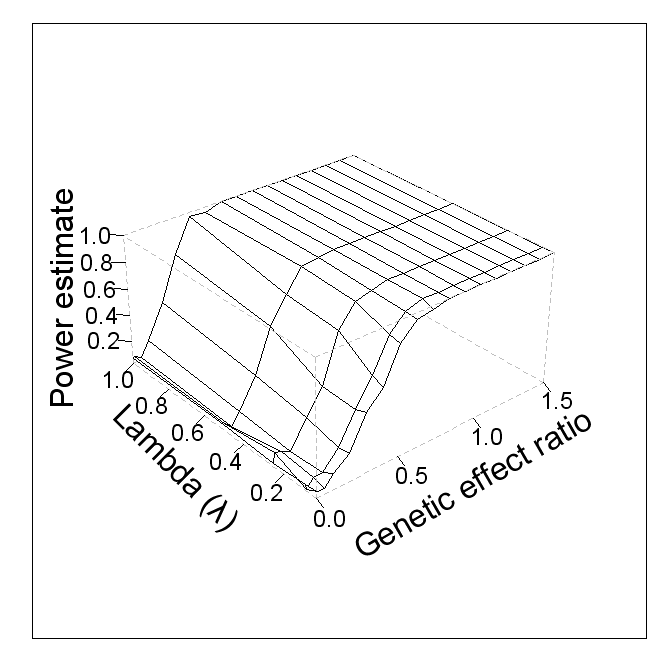


S


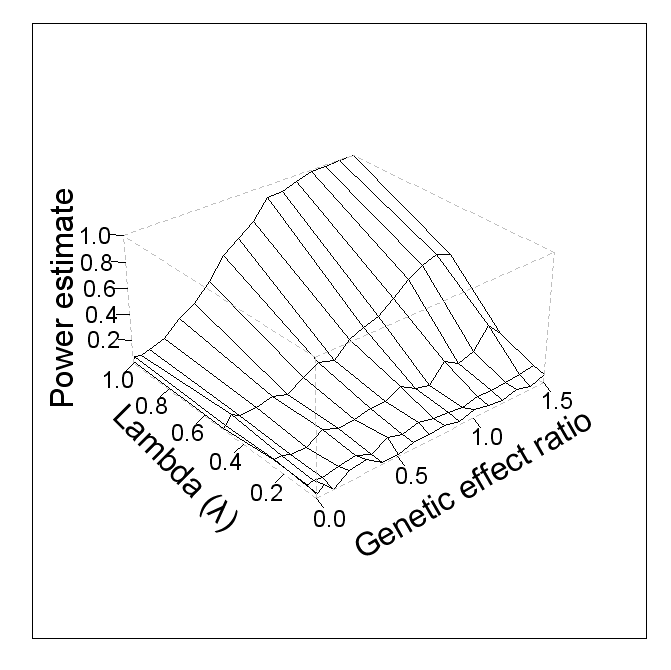


S x E

E. h^2^=0.25; q=0.25


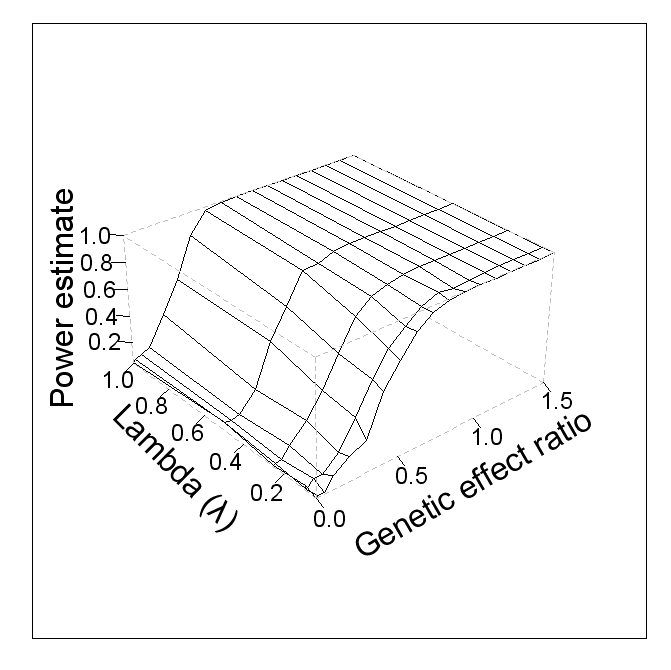


S


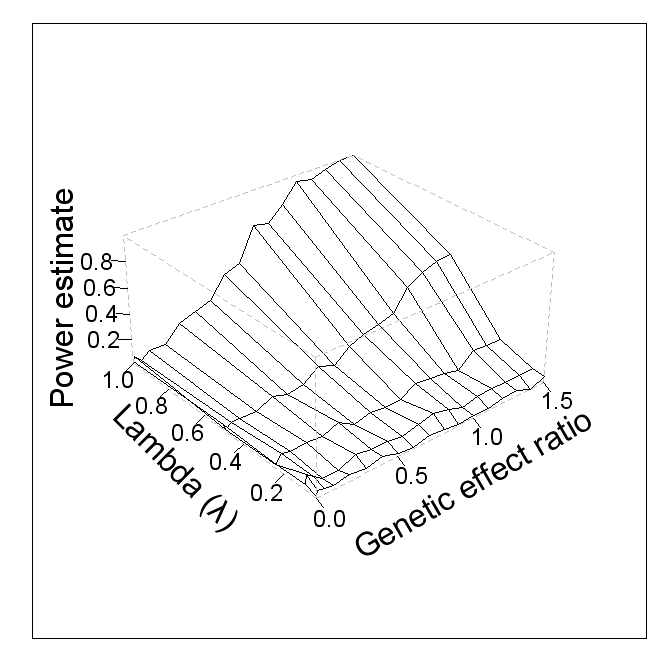


S x E

F. h^2^=0.25; q=0.05


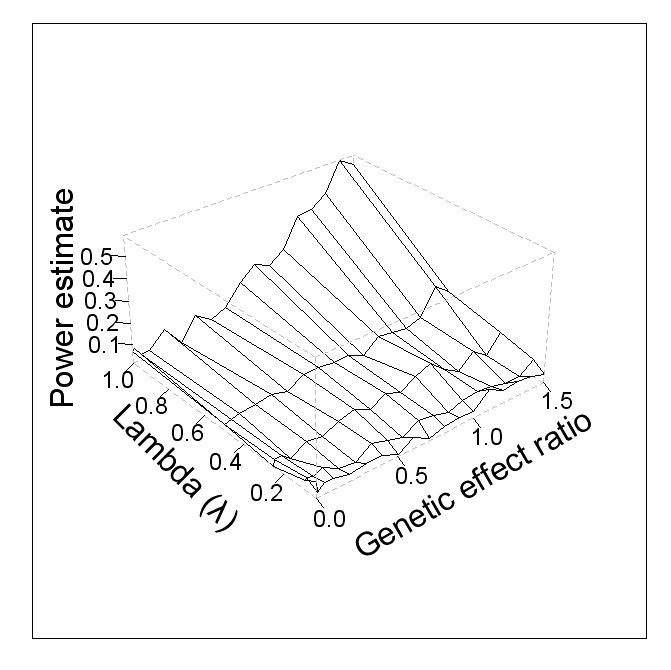


S x E


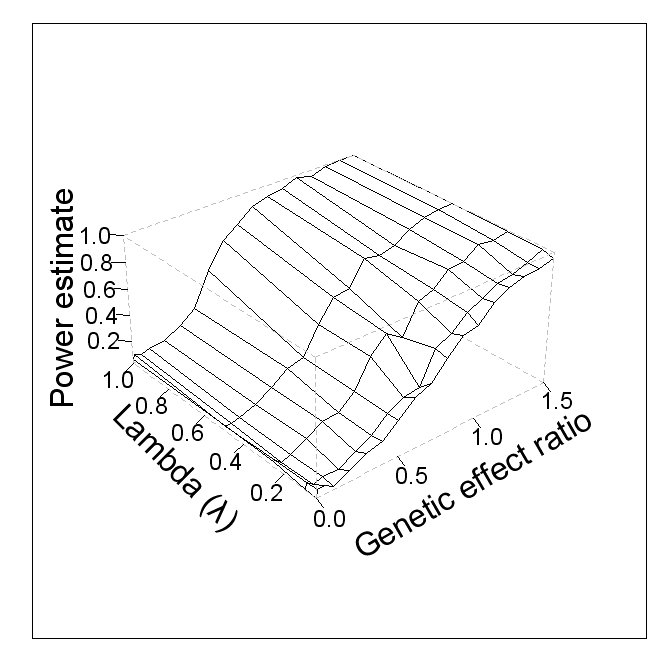


S

**Figure S1** Power of mixed linear model to detect phenotypic effects including gene by environment interaction. Pearl millet and maize samples, additional parameters. Data were simulated (see main text for details) considering the main effect (S) of a SNP, as well as the interaction of this SNP with environment (S x E). The simulated datasets were analyzed using a mixed linear model. The power was estimated as the ratio between simulated datasets for which a significant effect was detected and the total number of simulations. The results are presented as a function of the heritability (h^2^), allele frequency (q), genetic effect ratio (r) and the parameter modulating difference of SNP effects between environments (λ). The simulation was run for pearl millet (A, B, C) and maize (D, E, F).


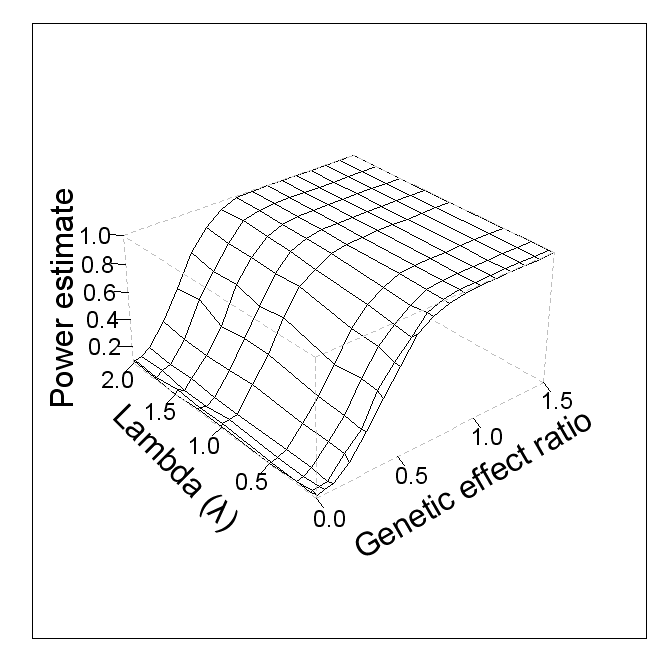


S

A. h^2^=0.75; q=0.25


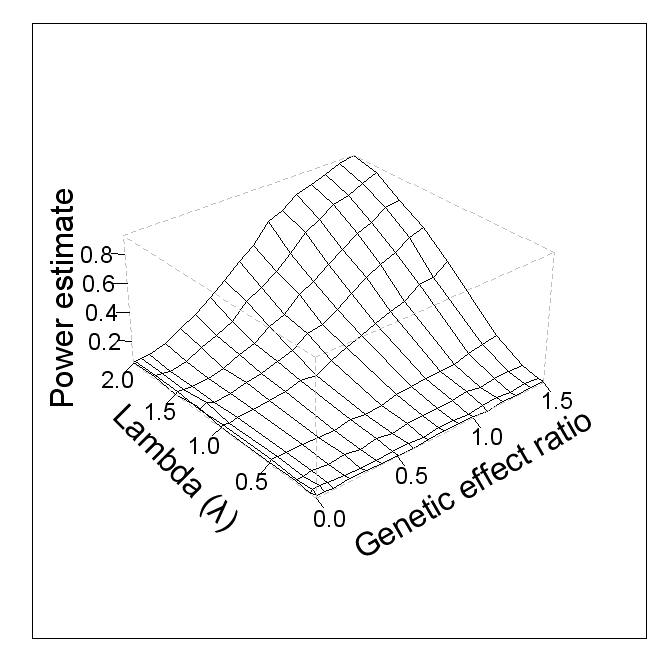


S x E


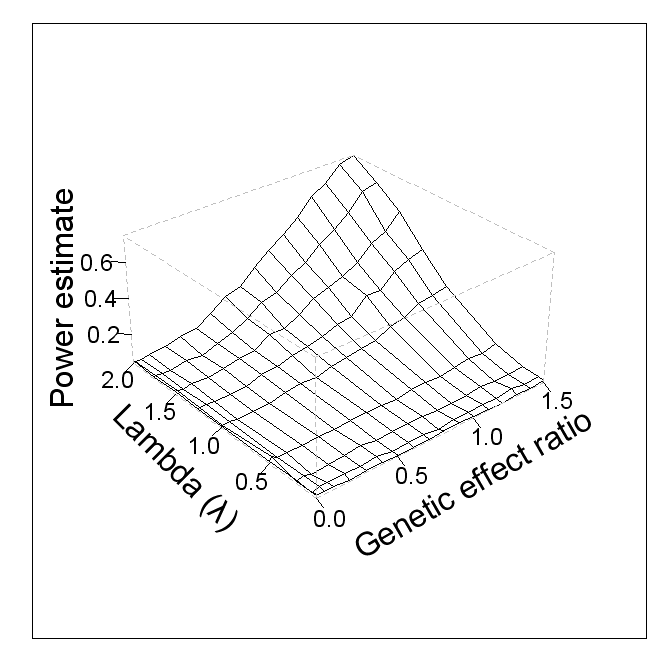


Q x S


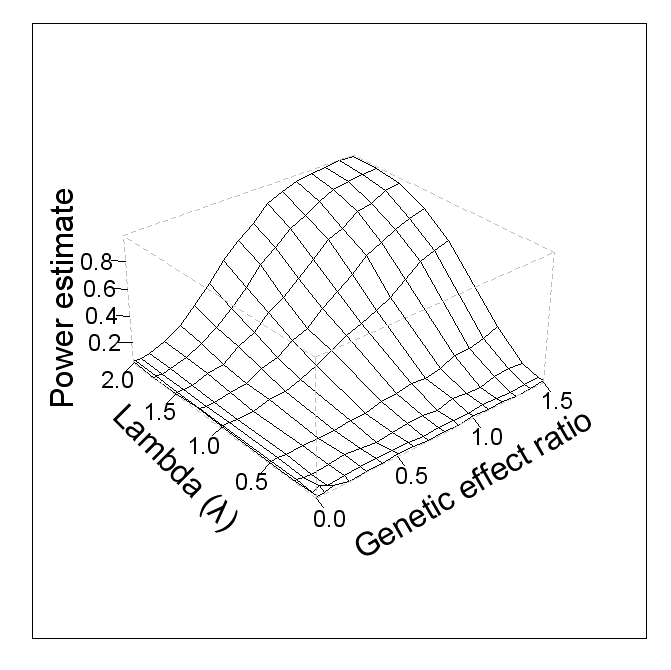


Q x S x E

X E

B. h^2^=0.25; q=0.5


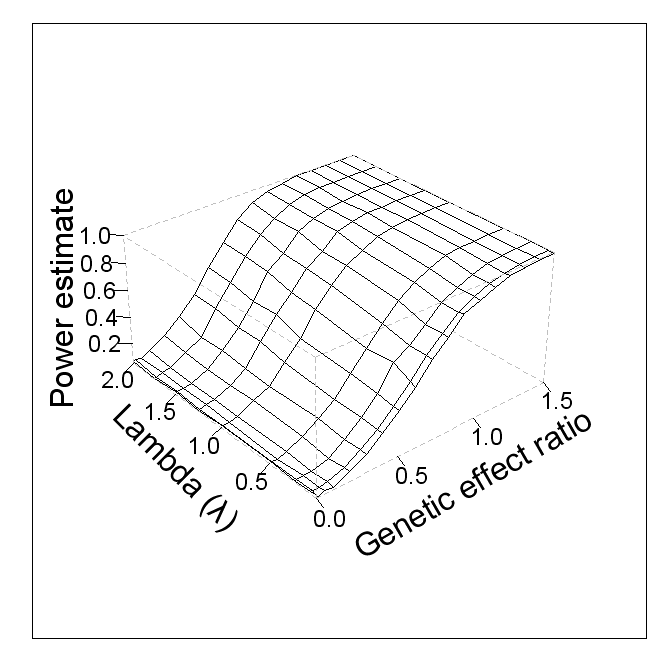


S


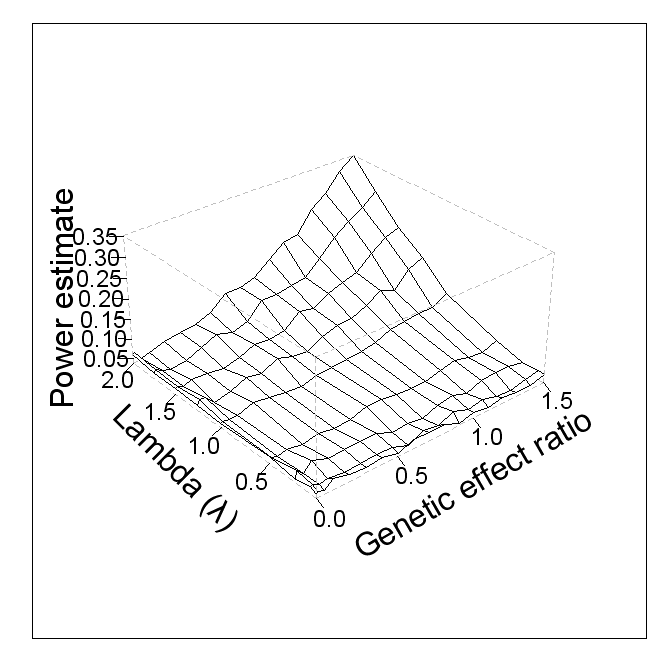


S x E


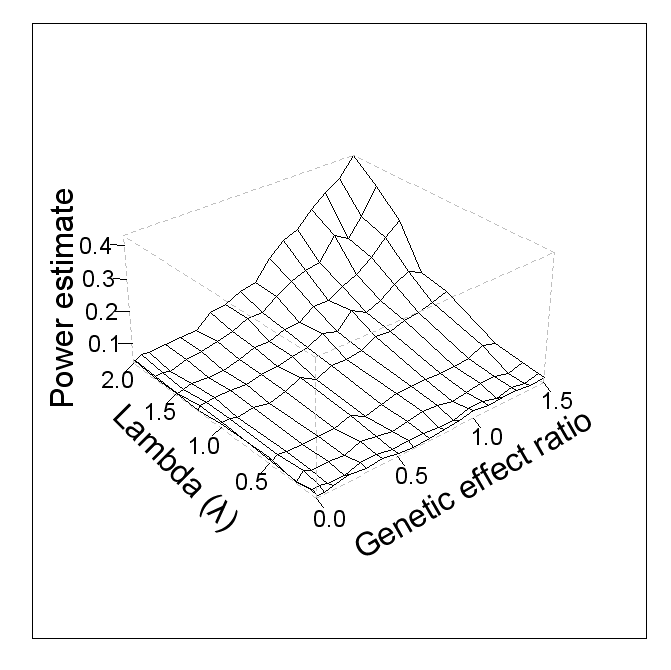


Q x S


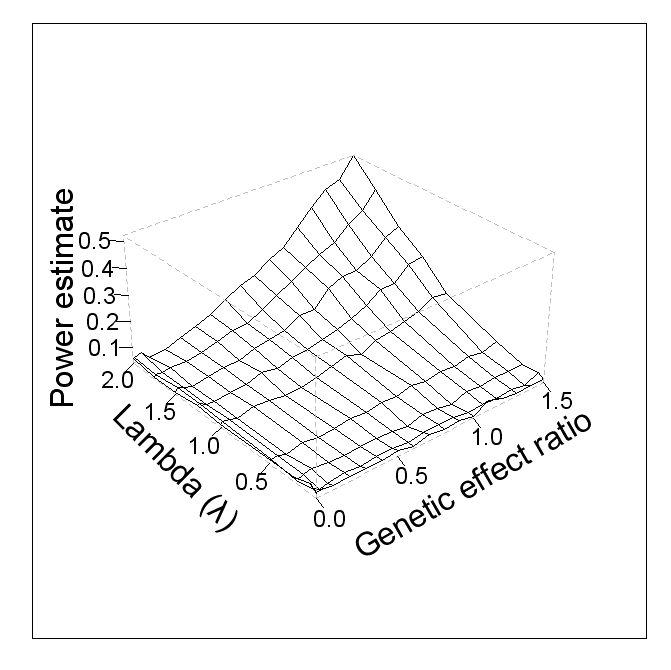


Q x S x E


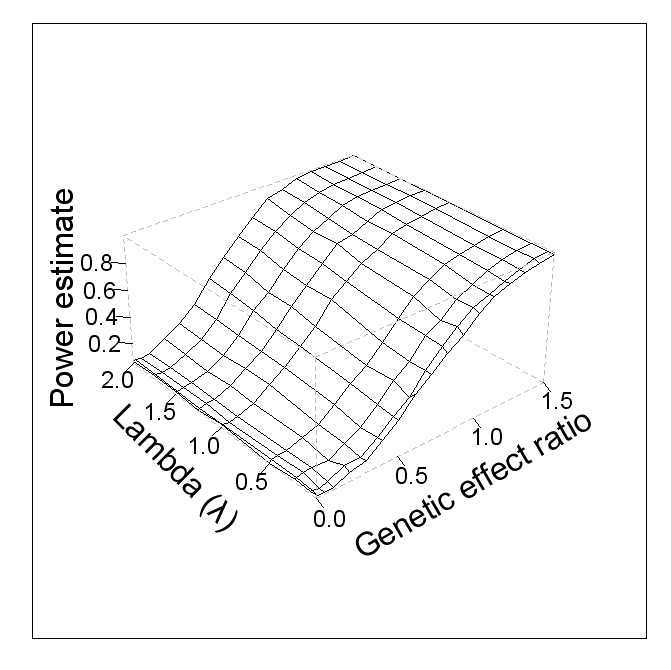


S


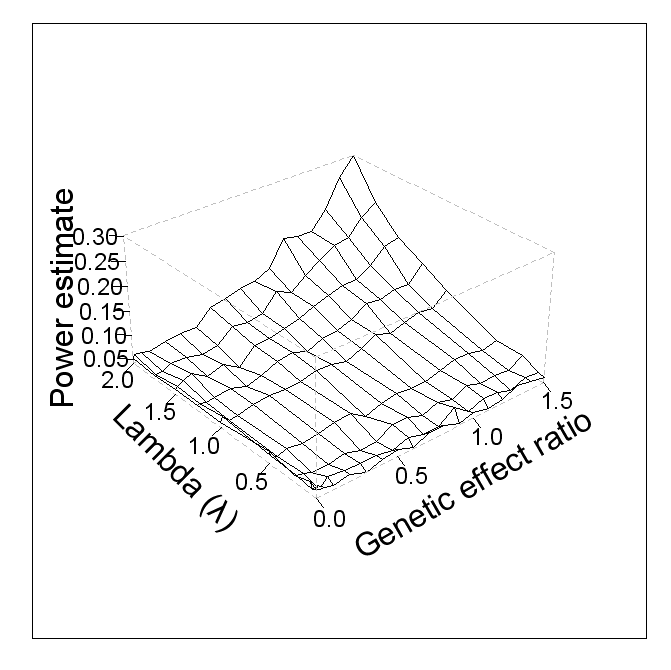


S x E


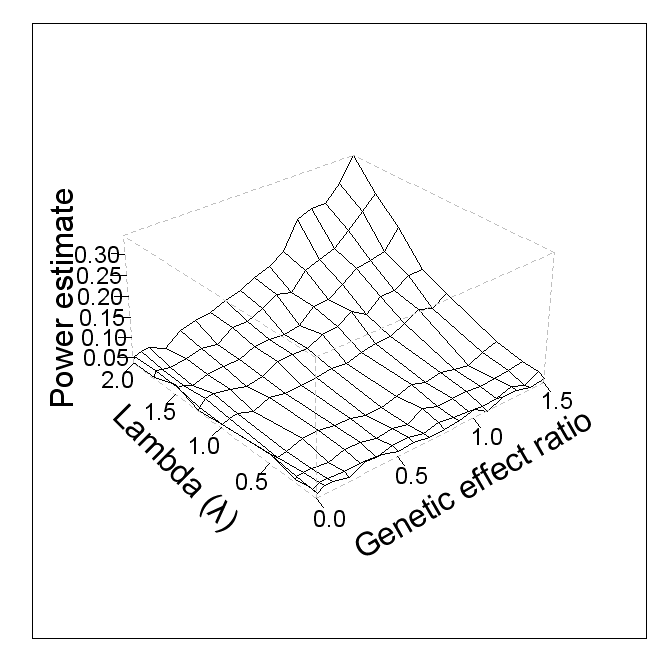


Q x S


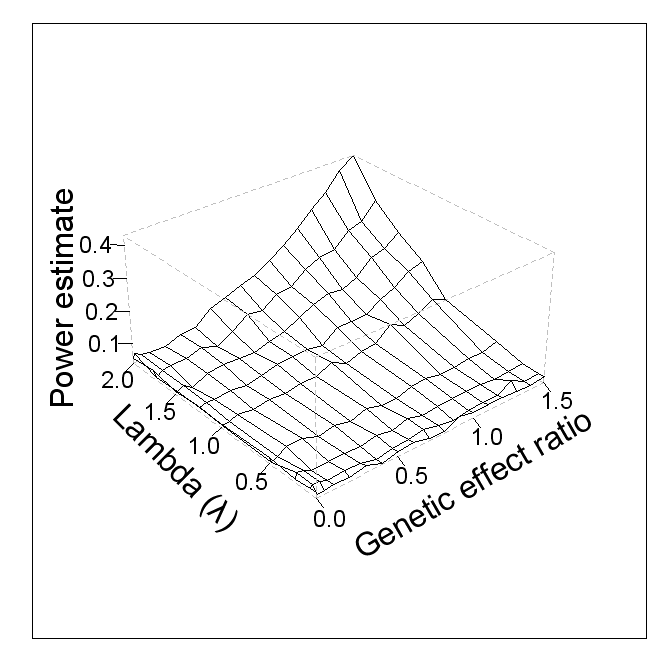


Q x S x E

C. h^2^=0.25; q=0.25

**Figure S2** Power of mixed linear model to detect phenotypic effects including 3-way interactions in the pearl millet sample. The data were simulated with 2 and 3-way interactions (see text, simulation scheme 3). The set of simulated effects includes SNP main effect (S), SNP by environment interaction (S x E), ancestry by SNP interaction (Q x S) and 3-way interaction between ancestry, SNP, and environment (Q x S x E). The proportion of simulations for which the effects were significantly detected by the statistical model is given according to heritability (h^2^), allele frequency (q), genetic effect ratio (r) and a parameter modulating effect across environments (λ).


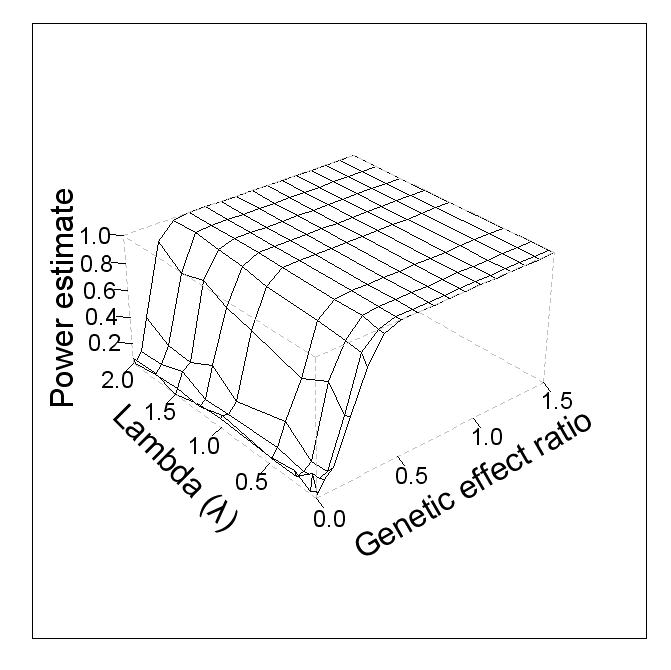


S


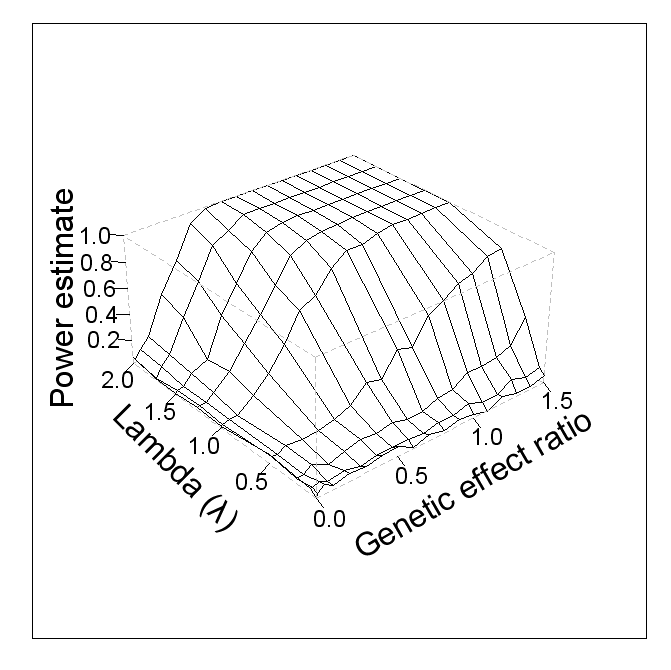


S x E


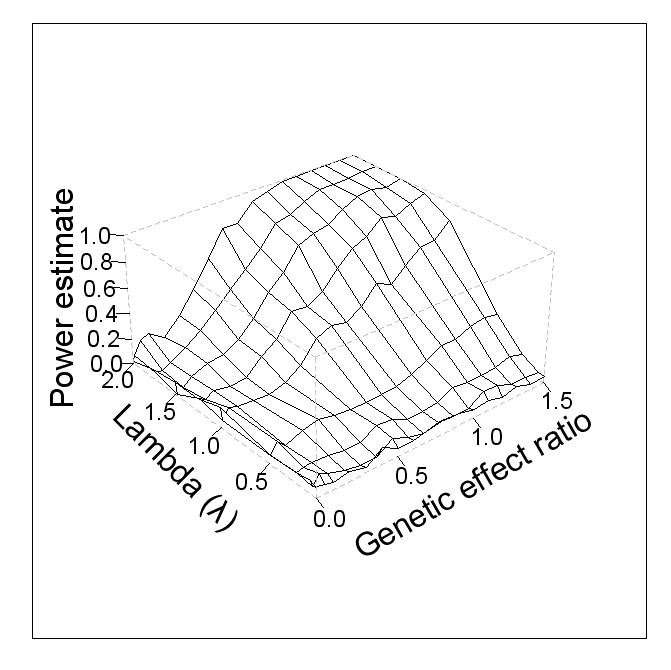


Q x S


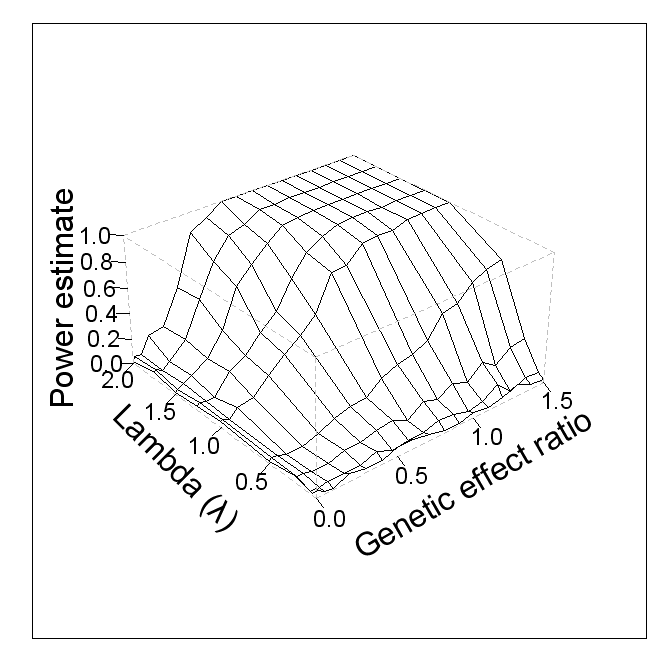


Q x S x E

x

A. h^2^=0.75; q=0.25


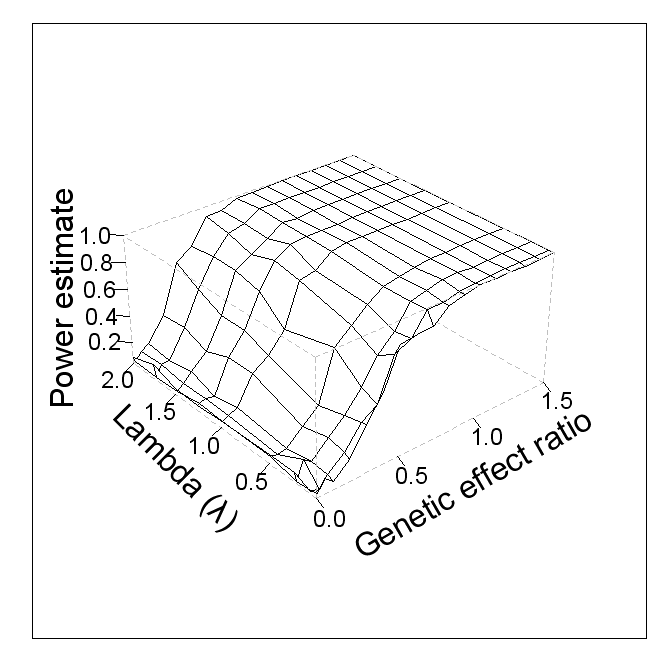


S

B. h^2^=0.75; q=0.05


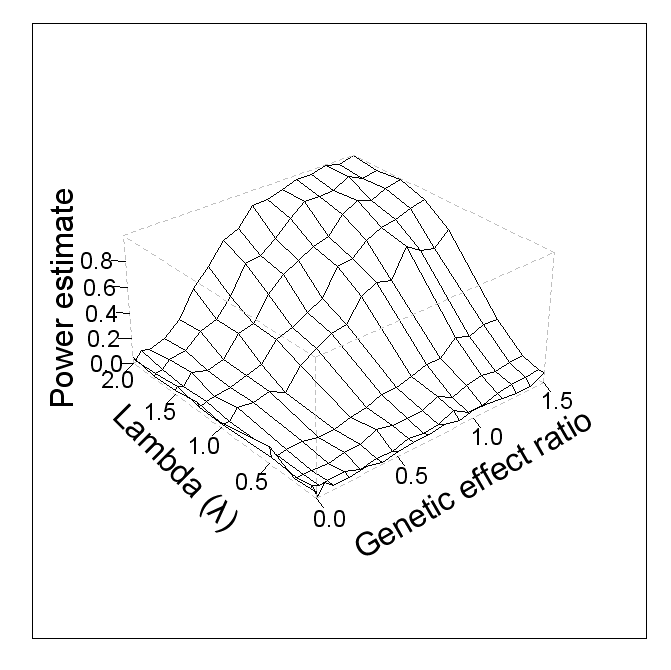


S x E


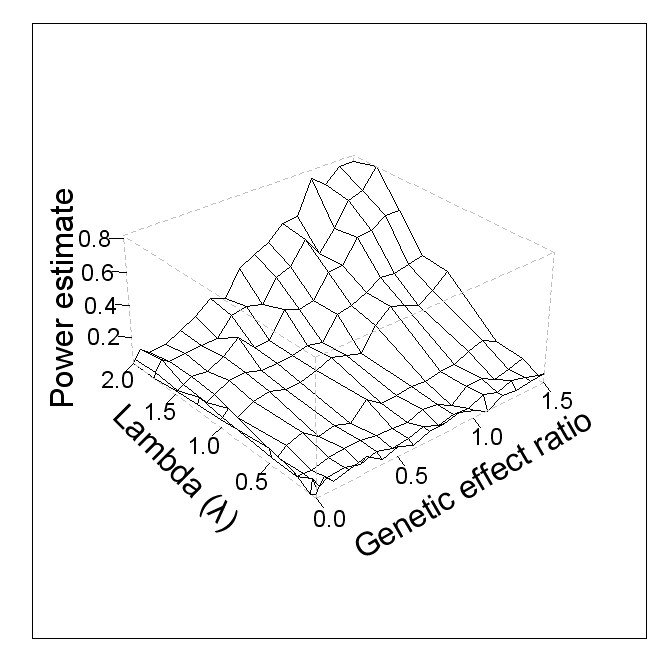


Q x S


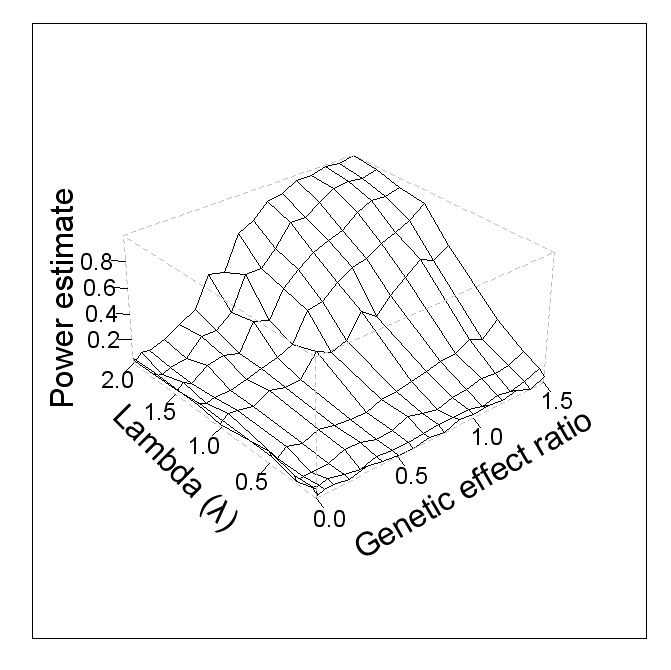


Q x S x E


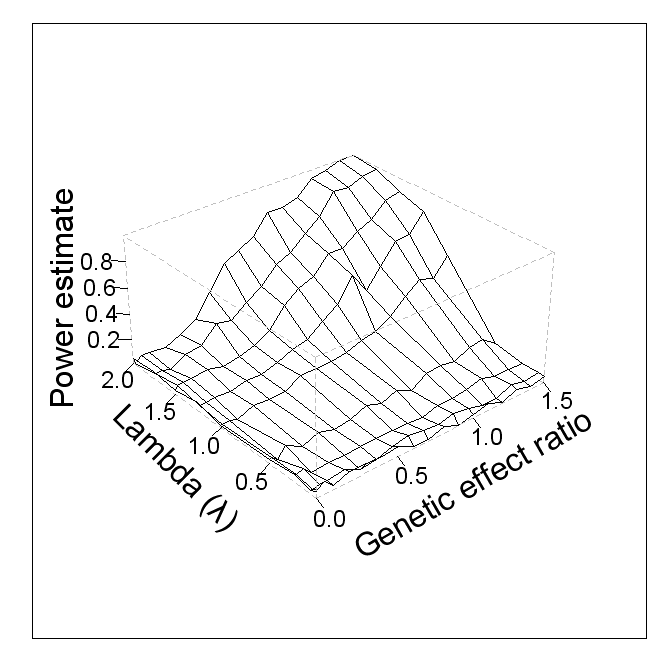


Q x S x E

C. h^2^=0.25; q=0.5


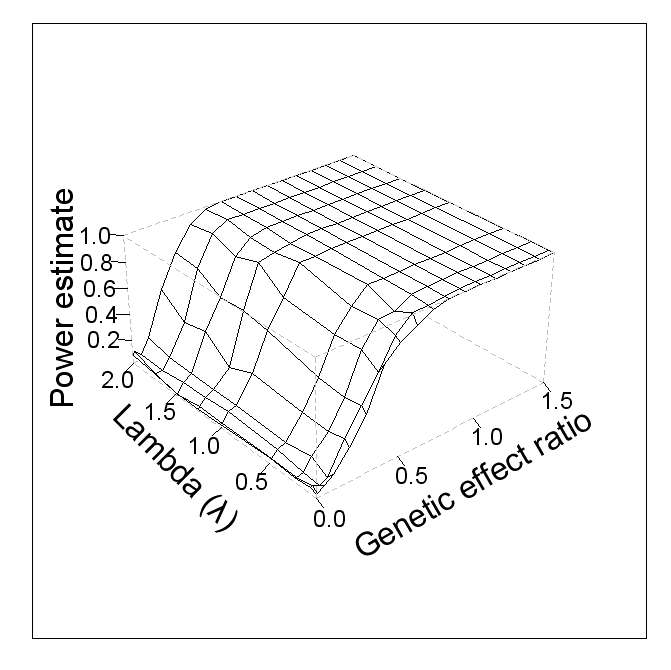


S


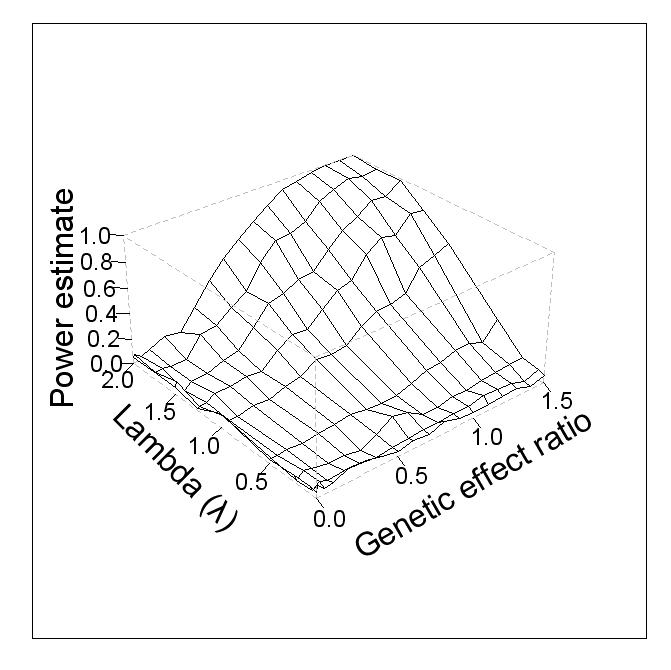


S x E


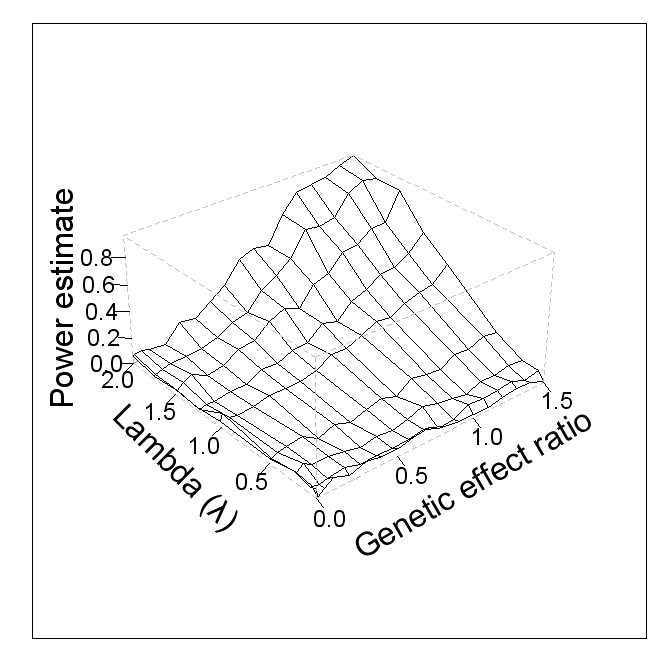


Q x S

E. h^2^=0.25; q=0.05


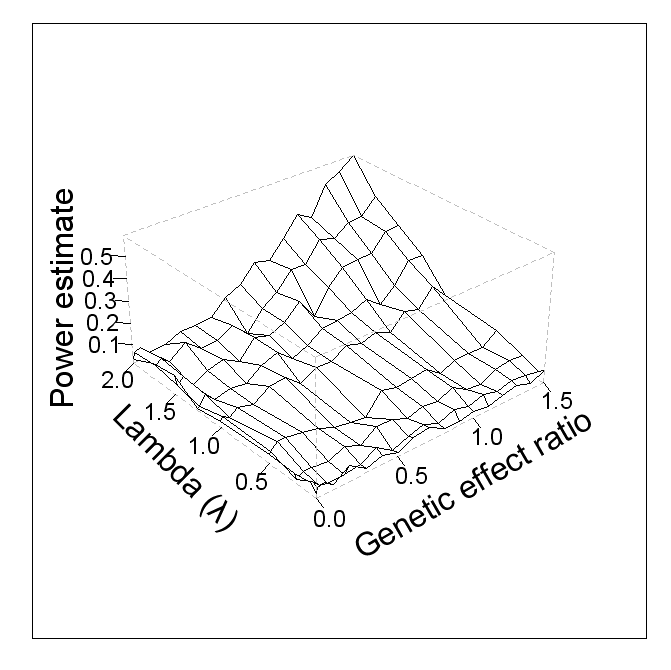


S x E


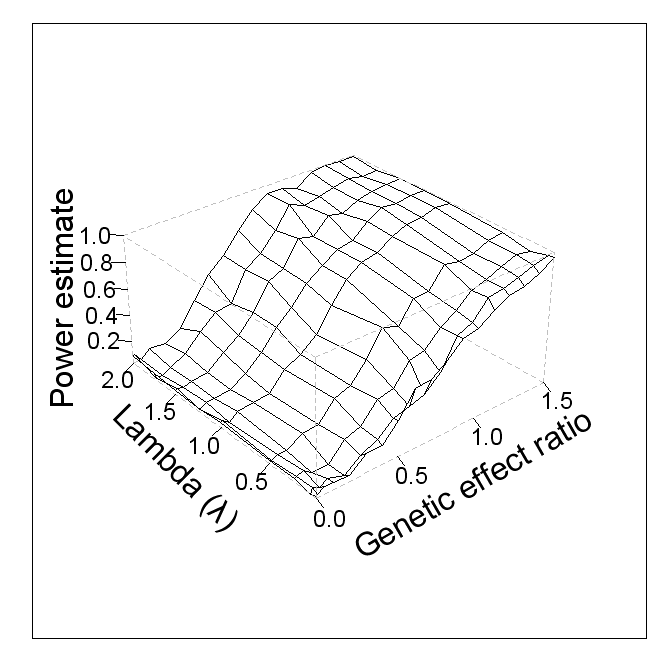


S


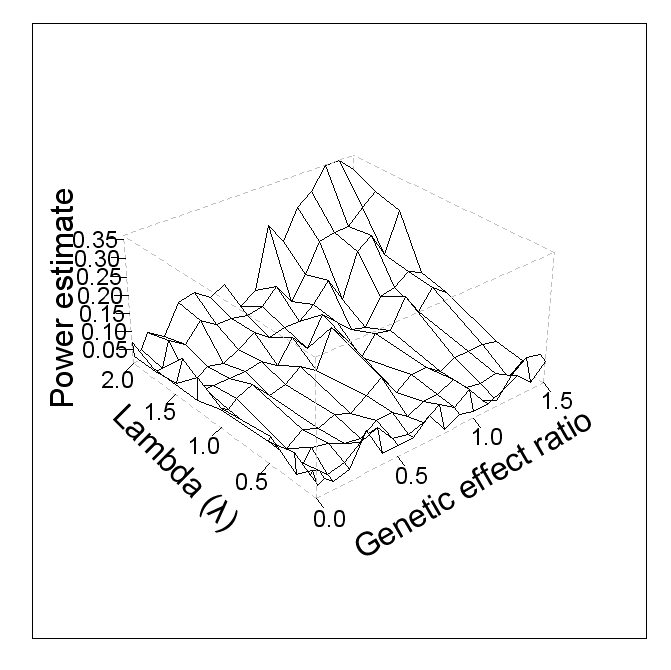


Q x S


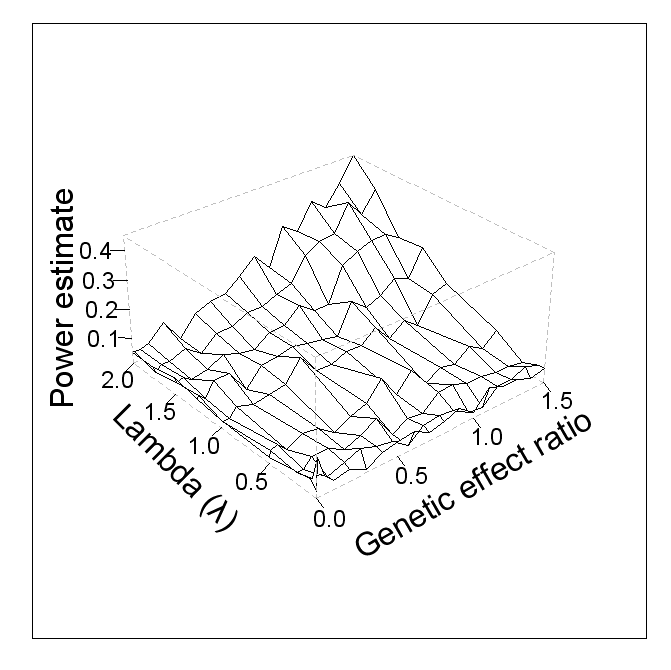


Q x S x E

D. h^2^=0.25; q=0.25


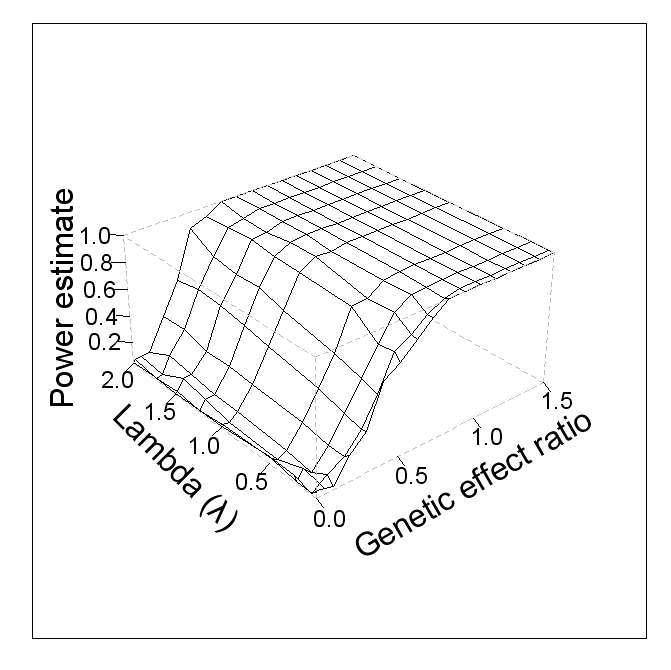


S


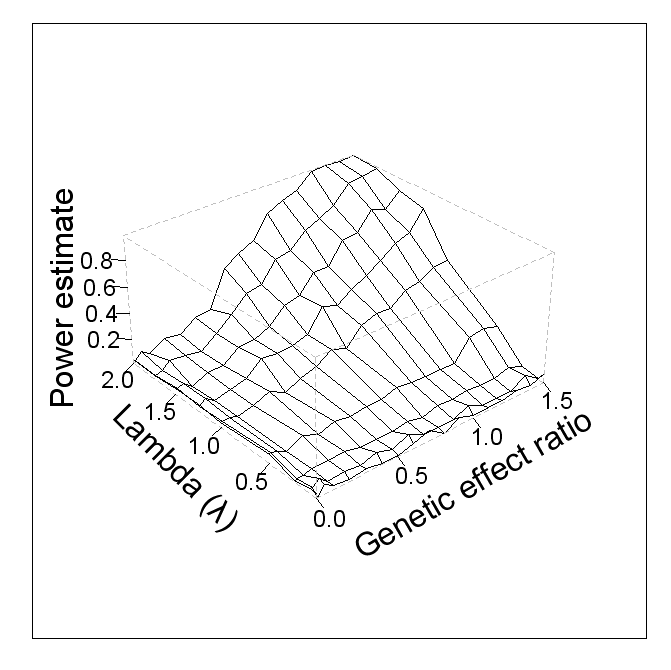


S x E


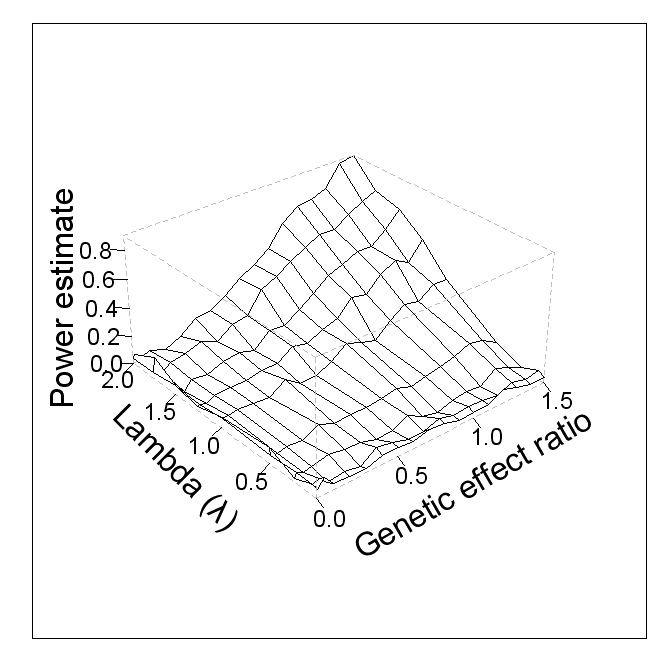


Q x S


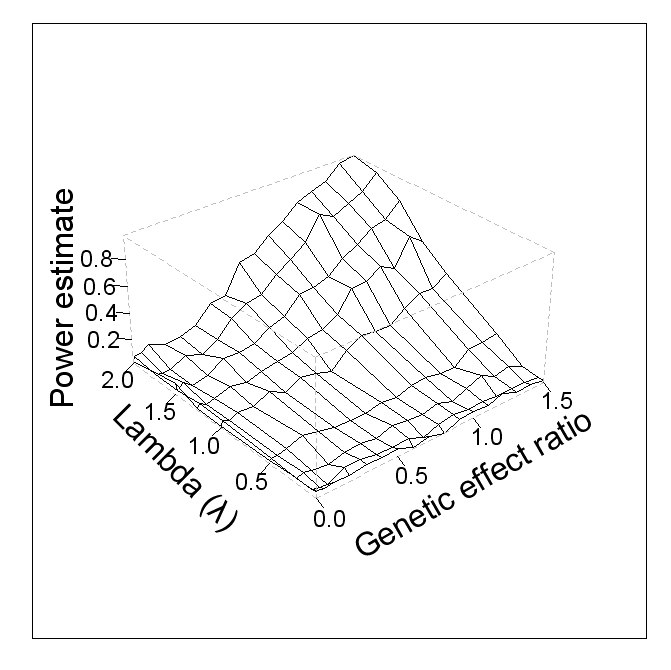


Q x S x E

**Figure S3.** Power of mixed linear model to detect phenotypic effects including 3-way interactions in the maize sample. The data were simulated considering SNP main effect (S), SNP by environment interaction (S x E), ancestry by SNP interaction (Q x S) and 3-way interaction between ancestry, SNP, and environment (Q x S x E). The proportion of simulations in which each effect was detected by the model is given, according to heritability (h^2^), allele frequency (q), genetic effect ratio (r) and a parameter modulating the effects across environments (λ).

A

B

C

D

**Figure S4** Model selection for the analysis of associations in a multi-trial design. A set of five nested models from the simplest main effect model (Fit1) to a 3-way interaction model (Fit5) were fitted. The five models were compared using AICC (left) and BIC (right) for pearl millet (A, B) and maize (C, D).

**Figure S5.**  Effect estimates of the *Vgt1* locus on days to silk for each of the 7 environments.

Effect estimate (grey rectangles) was calculated based on the maize inbred panel using the mixed linear model. Vertical lines represent 1.96-fold the standard error of each effect estimate. Significant effects (P<0.05) and non-significant effects (NS) are indicated. The significance results shown here are based on the original phenotypic data and were similar to those of the data resulting from Box-Cox transformation.


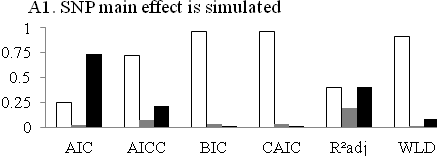

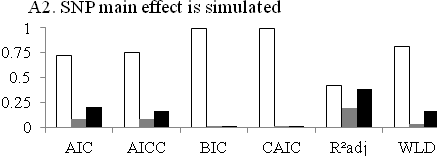

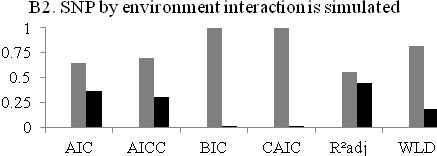

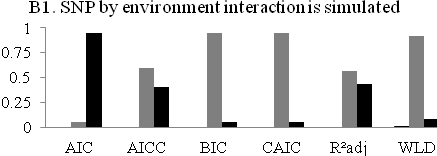

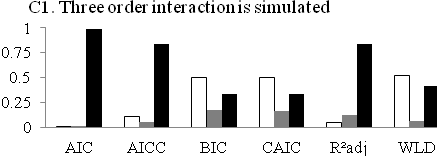

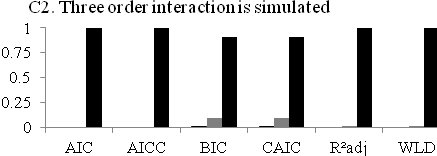


A. Pearl millet, n=90

B. Maize, n=277

Main effect model

SNP by environment interaction model

Three way interaction model

**Figure S6** Model selection based on different criteria.

Data were simulated with SNP main effect (A1- A2), SNP by environment interaction (B1-B2) and up to 3-way interactions between SNP, ancestry and environment (C1-C2). The simulation was based on original datasets from pearl millet (A1, B1, C1) and maize (A2, B2, C2). Each dataset was fitted to three competing models with different fixed parameters, and the models were compared using AIC, AICC, BIC, CAIC, R^2^_adj_ and WLD. The frequency of selection of each competing model is given for one combination of parameter values (q=0.5, h*^2^*=0.75, r=1, λ=1; see text for details). With respect to the simulation schemes used to generate the data, the expected adequate model is respectively the main effect model in white (A), the gene by environment interaction model in grey (B), and the 3-way interaction model in black (C).

Main effect model

Gene by environment interaction model

Three way interaction model

**Figure S7** Frequency of selection of competing models as a function of sample size in maize.

Datasets with 3-way interactions were generated (see text). In each run, three competing models were fitted to the simulated dataset: the simple main effect model, the gene by environment interaction model and the 3-way interaction model. Five criteria for model selection were used to compare the competing models: AIC, AICC, BIC, CAIC and R^2^_adj_ (two variants are considered for all criteria except AIC, see text for details). For each criterion, the frequency of selection of each model over the total number of simulations is given. The data were based on the maize inbred lines panel and used different sampling size (from n=90 to 240, see supplementary file). Inbred lines were randomly sampled among the 277 inbreds comprising the whole panel. The detection of the right model increased with sample size, particularly for BIC, BIC*, CAIC and CAIC*. This underlined the importance of sample size for the performance of model selection under REML.
