# Supplementary material for: Association studies including genotype by environment interactions: prospects and limits
Source: BMC Genet. 2014 Jan 6;15:3. doi: 10.1186/1471-2156-15-3 (PMC3901036; doi:10.1186/1471-2156-15-3)
Supplement: Additional file 2: Table S1 — Minimum effect detected with a power of 95% in the model with gene by environment interaction. Table S2 Expectation of minimum effect detected with a power of 95% in the model with three way interaction. Table S3 Model selection based on WLD. Table S4 Wald test for Vgt1 effect analyzed independently into each trial. Table S5 Formulation of information criteria. Table S6 Rate of selection of competing models by information criteria, at different values of effect size parameter r. Table S7 Significance of the effect of data parameters on the rate of success of information criteria. [file 1471-2156-15-3-S2.doc]

**Saïdou *et al.***

**Supplementary tables**

**Table S1** Minimum effect detected with a power of 95% in the model with gene by environment interaction.

| Parameters | | | Effects | |
| --- | --- | --- | --- | --- |
| Sample | h^2^ | q | S | S x E |
| Pearl millet (n=90) | 0.75 | 0.5 | 4.61 | 4.44 |
|  |  | 0.25 | 5.12 | 5.46 |
|  |  | 0.05 | 12.29 | >10.25^a^ |
|  | 0.25 | 0.5 | 7.68 | >10.25^b^ |
|  |  | 0.25 | 9.10 | >10.25^c^ |
|  |  | 0.05 | >15.37^d^ | >10.25^e^ |
| Maize (n=277) | 0.75 | 0.5 | 3.27 | 3.05 |
|  |  | 0.25 | 3.58 | 3.92 |
|  |  | 0.05 | 6.87 | 9.6 |
|  | 0.25 | 0.5 | 5.23 | 10.46 |
|  |  | 0.25 | 6.54 | 11.34 |
|  |  | 0.05 | 12.75 | >13.08^f^ |

Effects on the number of days to flowering were simulated. For each effect, the minimum effect size detected in 95% of iterations is given. When the maximum effect simulated for a given term was detected in less than 95% of iterations, a footnote gives the actual power reached for the considered effect size (^a^ 0.904; ^b^ 0.803; ^c^ 0.705; ^d^ 0.832; ^e^ 0.274; ^f^ 0.55).

**Table S2** Expectation of minimum effect detected with a power of 95% in the model with three way interaction.

| Parameters | | | Effects | | | |
| --- | --- | --- | --- | --- | --- | --- |
| Sample | h^2^ | q | S | S x E | Q x S | Q x S x E |
| Pearl millet (n=90) | 0.75 | 0.5 | 4.67 | 4.97 | >2.87^a^ | 3.45 |
|  |  | 0.25 | 5.1 | >5.74^b^ | >2.87^c^ | 4.59 |
|  | 0.25 | 0.5 | 7.65 | >5.74^d^ | >2.87^e^ | >5.74^f^ |
|  |  | 0.25 | 8.94 | >5.74^g^ | >2.87^h^ | >5.74^i^ |
| Maize (n=277) | 0.75 | 0.5 | 2.94 | 3.2 | 3.2 | 3.85 |
|  |  | 0.25 | 3.53 | 3.74 | 3.85 | 4.27 |
|  |  | 0.05 | 6.83 | 9.4 | >5.61^j^ | 11.11 |
|  | 0.25 | 0.5 | 5.36 | 9.61 | 6.41 | 11.22 |
|  |  | 0.25 | 6.48 | 10.25 | >6.41^k^ | 12.82 |
|  |  | 0.05 | 13.03 | >12.82^l^ | >6.41^m^ | >12.82^n^ |

aa: 0.921 bb: 0.924 cc: 0.744 dd: 0.353 ee: 0.429 ff: 0.52 gg: 0.302 hh: 0.344 ii: 0.428 jj: 0.82 kk: 0.9 ll: 0.59 mm: 0.32 nn: 0.45

Effects on the number of days to flowering were simulated. For each effect, the minimum size detected in 95% of iterations is given. When the maximum effect simulated for a given term was detected in less than 95% of iterations, a footnote gives the actual power reached for the considered effect size : ^a^ 0.921; ^b^ 0.924; ^c^ 0.744; ^d^ 0.353; ^e^ 0.429; ^f^ 0.52; ^g^ 0.302; ^h^ 0.344; ^i^ 0.428; ^j^ 0.82; ^k^ 0.90; ^l^ 0.59; ^m^ 0.32; ^n^ 0.45.

.

**Table S3** Model selection based on WLD

|  | Fit5 | Fit4 | Fit3 | Fit2 | Fit1 | Selected  model |
| --- | --- | --- | --- | --- | --- | --- |
| Specific terms | Q_i_ x S x E | Q_i_ x S | S x E | Q_i_ x E | S |  |
| **Pearl millet** | | | | | | |
| *N* | *-* | *6* | *6* | *1* | *6* |  |
| *PHYC* (101) | 0.0537 | 0.1462 | 0.0717 | 0.1231 | 0.0045 | Fit5 |
| *PHYC* (128) | 0.1049 | 0.1763 | 0.3436 | 0.1229 | 0.0093 | Fit5 |
| *PHYC* (155) | 0.1371 | 0.1511 | 0.0472 | 0.1224 | 0.0007 | Fit3 |
| *PHYC* (456) | 0.229 | 0.1374 | 0.167 | 0.1261 | 0.001 | Fit5 |
| *PHYC* (615) | 0.1329 | 0.0555 | 0.3591 | 0.1225 | 0.0153 | Fit5 |
| *PHYC* (645) | 0.229 | 0.1374 | 0.167 | 0.1261 | 0.001 | Fit5 |
| *PHYC* (697) | 0.237 | 0.152 | 0.5352 | 0.1259 | 0.0015 | Fit5 |
| **Maize** | | | | | | |
| *N* | *-* | *2* | *2* | *1* | *2* |  |
| *Vgt1* | 0.1897 | 0.3125 | 2 x 10^-06^ | < 2 x 10^-26^ | 0.3824 | Fit3 |

Wald test was used to compare the five models fitted to flowering time data in pearl millet and maize respectively. Full description of the models is available in the text. Descending model simplification is carried out, so each model is compared to the nearest small model. For example, Fit5 is compared to Fit4 by removing the terms corresponding to three order interactions (Q_i_ x S x E). The number of removed terms into each step is given (N). The large model is conserved when at least one of the N specific terms in this model is significant (*P* < 0.05). The achievement of this condition means that the minimum P-value across the N specific terms is significant. So we reported in the table this minimum P-value for the test of each model (significant P-value are highlighted). SNP position for *PHYC* is indicated in brackets (first column). Note that the number of terms N corresponds also to the number of tests carried out to compare the two models in each step, and this illustrates the multiple testing issues in this procedure (19 tests per marker for pearl millet and 7 tests for maize marker).

**Table S4** Wald test for *Vgt1* effect analyzed independently into each trial.

| Environment code | Df | Transformed data | | | Original data | | |
| --- | --- | --- | --- | --- | --- | --- | --- |
|  |  | Sum of squares | Wald statistic | P-value | Sum of squares | Wald statistic | P-value |
| 11 | 1 | 74.50 | 5.18 | 0.0229 | 66.53 | 4.61 | 0.0317 |
| 12 | 1 | 48.69 | 5.38 | 0.0203 | 43.43 | 5.36 | 0.0206 |
| 13 | 1 | 38.58 | 2.80 | 0.0940 | 47.78 | 2.8 | 0.0941 |
| 14 | 1 | 37.01 | 5.15 | 0.0232 | 37.38 | 5.12 | 0.0237 |
| 15 | 1 | 69.04 | 4.19 | 0.0406 | 62.35 | 3.79 | 0.0515 |
| 17 | 1 | 10.79 | 2.28 | 0.1310 | 11.82 | 2.29 | 0.1305 |
| 18 | 1 | 11.44 | 3.25 | 0.0713 | 10.78 | 3.23 | 0.0723 |

The association between *Vgt1* and flowering time were assessed independently in each field trial using mixed linear model as Y_i_ = Q_p_ + S_i_ + K + e, where Q_p_ is the fixed effect of the ancestry in population P, S_i_ is fixed effect of *Vgt1* allele hold by individual i, and K modulates the variance covariance structure based on kinship matrix; e is the residual of the model. Environment code refers to the location in which each trial was performed. The results are presented respectively for data transformed using Box-Cox transformation (transformed data) and for original data (i.e. non-transformed data). Combining individual probabilities using Fisher’s method over the 7 trials led to a Chi square probability of 7.52 x 10^-5^ for transformed data, and 1.18 x 10^-4^ for original data. Thus, the effect of *Vgt1* is not significant in certain trials considered individually, but the global effect across trials is significant. Df: degree of freedom for *Vgt1* effect.

**Table S5** Formulation of information criteria

| Criterion | Formula | Notation |
| --- | --- | --- |
| AIC | −2l + 2s | AIC |
| AICC | −2l + 2s m / (m−s−1) | AICC |
|  | −2l + 2s (N−p) / (N−p−s−1) | AICC* |
| BIC | −2l + s log(m) | BIC |
|  | −2l + s log(N−p) | BIC* |
| CAIC | −2l + s log(m+1) | CAIC |
|  | −2l + s log(N−p+1) | CAIC* |
| R^2^_adj_ | 1 − (1−R^2^) N / (N−p) | R^2^ _adj_ |
|  | 1 − (1−R^2^) N/(N−p−k) | R^2^_adj_* |

The maximum log likelihood is noted l; p is the number of fixed parameters in the model, k is the number of variance parameters, s is the total number of parameters (i.e. s=p+k), m is the number of individuals (or *statistical units*), N is the total number of observations (product of the number of individuals by the number of repeats). Log function is natural logarithm. R^2^ is the squared correlation coefficient calculated as
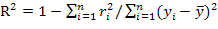
; where *r_i_* is the conditional residual for the ith observation, calculated as the difference between the observed value and the predicted value. For all criteria but AIC, two variants are defined, with respect to the way of accounting for sample size. A star (*) is used in this paper to distinguish these two variants.

**Table S6** Rate of selection of competing models by information criteria, at different values of effect size parameter r.

Table S9 is available as Excel file.

**Table S7** Significance of the effect of data parameters on the rate of success of information criteria

| Criterion | Intercept | Sample size | σ_G_ | q_0_ |
| --- | --- | --- | --- | --- |
| AIC | 0.073 | 0.75 | **4.42 x 10^-08^** | 0.74 |
| AICC | **3.52 x 10^-07^** | **2.4 x 10^-46^** | **8.92 x 10^-20^** | 0.17 |
| AICC* | **8.5 x 10^-4^** | **1.38 x 10^-10^** | **7.28 x 10^-13^** | 0.45 |
| BIC | **1.59 x 10^-38^** | **<10^-324^** | **3.81 x 10^-44^** | **2.70 x 10^-08^** |
| BIC* | **8.68 x 10^-50^** | **<10^-324^** | **5.28 x 10^-42^** | **1.39 x 10^-07^** |
| CAIC | **2.86 x 10^-38^** | **<10^-324^** | **9.71 x 10^-44^** | **4.79 x 10^-08^** |
| CAIC* | **1.45 x 10^-49^** | **<10^-324^** | **9.67 x 10^-42^** | **1.89 x 10^-07^** |
| R^2^_adj_ | **3.68 x 10^-3^** | **3.07 x 10^-246^** | **2.25 x 10^-3^** | **9.45 x 10^-3^** |
| R^2^_adj_* | **2.28 x 10^-3^** | **1.03 x 10^-249^** | **1.36 x 10^-3^** | **8.4 x 10^-3^** |

Data were simulated with three way interaction (simulation scheme 3) on maize sample and fitted by 3 competing models. Subsets of different sizes were sampled (from n=90 to n=240) and 10 random sampling were performed for each sample size. The rate of success (frequency of selection of the three way interaction model) was recorded in each single run (one run consisted of 1000 iterations of data simulation and model selection using the same subset). We then analyzed the distribution of the resulting rate of success using the generalized linear model (GLM) y = µ + α_i_ + β_j_ + λ_l_, were µ is the intercept, α_i_ is the effect of sample size level i, β_j_ is the effect of trait standard deviation (σ_G_) in the run j, and λ_l_ is the effect of average ancestry (q_0_) in the sampled subset l. As the rate of success y is a proportion, GLM was set with a logit link function (R, version 2.7.2). The p-value of significance of each term is given in the table, with respect to the criterion used for model selection. Significant p-values are highlighted (P<0.05).
