# Supplementary material for: Association studies including genotype by environment interactions: prospects and limits
Source: BMC Genet. 2014 Jan 6;15:3. doi: 10.1186/1471-2156-15-3 (PMC3901036; doi:10.1186/1471-2156-15-3)
Supplement: Additional file 3 — Assessment of methods for model selection. [file 1471-2156-15-3-S3.docx]

**Saïdou et *al.***

**Supplementary File**

**Assessment of methods for model selection**

A commonly used approach for model selection consists in the selection of terms based on Wald test (WLD) or F test significance. In this approach, one possible way is to construct the full model with the largest set of terms (main factors and their interactions) and to progressively remove non-significant terms (backward model selection). The inverse way consists in adding terms progressively in the model based on their significance (forward model selection). This approach is commonly admitted with the simplest designs, for example in association tests based on a single trait and a single environment. But for the more complex designs (multi-trait or multi-environment), this approach presents shortcomings. For instance, the number of tests is generally high, because one test is required for each added or removed single term. This multiple testing inflates type I error as the number of additional terms in the full model compared to the simplest models is generally large in this context. Also, the order of inclusion or exclusion of terms in the model does generally matter in these approaches. This could yield contrasting results, with a model selection not consistent according to the order of terms inclusion.

An alternative approach for model selection consists in the use of information criteria based on the log-likelihood, such as Akaike Information Criterion (AIC) [S1], or Bayesian Information Criterion (BIC) [S2]. This approach implies a limited number of tests. Only a single test is required to compare two or more models with these criteria, whatever the difference of terms between the two models. This ensures the best control of type I error with regard to the multiple testing issue, compared to the method based on Wald test.

Nonetheless, the use of information criteria to compare mixed models with different fixed effects is seen as inadequate by a part of the scientific community when mixed model is fitted by restricted maximum likelihood (REML) method [S3]. As REML is the better method for unbiased variance components estimation (in opposite to maximum likelihood), this method is generally preferred to fit mixed model.

So we used REML in this study, and we designed a simulation to assess empirically information criteria under REML. Datasets were simulated based on controlled parameters. Wald test based method (WLD) was also used as a benchmark to see if information criteria could provide a selection performance as high as that of WLD. This is carried out in order to prospect the possibility of taking benefit from information criteria as alternative to WLD method, to avoid the multiple testing issue.

**Method of data simulation and criteria assessment**

The performance of model selection was assessed for three set of methods: i) Wald test (WLD); ii) information criteria including AIC [S4], AICC [S1], CAIC [S5], BIC [S2], and iii) the predictive criteria R^2^_adj_ [S6]. Two variant formulations of each information criterion (except AIC) were considered (Table S5). Datasets were generated based on the three simulation schemes already described in the main text: (i) data pattern with no interaction (simulation scheme 1), (ii) gene by environment interaction (simulation scheme 2) and (iii) two to three way interactions (simulation scheme 3). A set of competing models was fitted to each simulated dataset, whatever its pattern. For simplicity’s sake, the set of models included only the three mixed linear models described in the text: the mixed model with no interaction (model 1), the mixed model with a gene by environment interaction (model 2) and the mixed model with two and three way interactions (model 3). These models were fitted under REML and are different with respect to the set of fixed effects. For each dataset, model selection criteria were used alternately to compare the competing models. The joint process of data simulation and model selection was iterated 1000 times for each combination of parameters. For this analysis, the parameters h^2^, q and λ did not vary and were fixed respectively at 0.75, 0.5 and 1. The effect ratio r varied from 0.1 to 1.5 (7 values) for all criteria but WLD (only r = 1 was tested for WLD). For each criterion, we then calculated the frequency with which each competing model was selected. This analysis was performed on both the pearl millet and maize panels and using the whole panels (n=90 and n=277 respectively).

The larger sample (maize) was also used to assess the impact of sample size on model selection in the case of a three-way interaction (simulation scheme 3). The sample size n was set at six equally spaced values between n=90 and n=240 (90, 120, 150, 180, 210 and 240). For each value, 10 subsets were randomly sampled from the whole sample of 277 lines. The simulation was incremented on these subsets to assess variations in the performance of the criteria with different sample size (r was set at 1 for this last analysis).

**Results**

**Performance of information criteria for model selection**

We tested the ability of different information criteria to select the right mean structure (i.e. the fixed effects parameters) for mixed linear model fitted by REML. Variance parameters were the same for all the compared models, so that the selection specifically targeted the set of fixed parameters. For each criterion, we computed the frequency of selection of each of the competing models for the pearl millet and maize datasets respectively (Figure S6 and Table S6). The appropriate structure of each simulated dataset was known beforehand because the data were simulated with explicit parameters. That being the case, the model with no interaction (model 1) was the best model for the data generated under simulation scheme 1; the gene by the environment interaction model (model 2) was the best model for data generated under simulation scheme 2 and the model with three way interaction (model 3) was the best model for data from simulation scheme 3. Across all schemes, the frequency of success of each information criterion (i.e. frequency of selection of the correct model) tended to increase when r increases (Table S6). In some cases, higher values of r led to a plateau at the maximum frequency of success. Relative to the range of values tested, r=1 represents an intermediate effect size. Figure S6 shows the performance of information criteria at r=1. The result for only one variant is plotted for each criterion, as the feature was similar between two variants of the same criterion (Table S6). In the pearl millet sample (Figure S6A), three categories of behaviour were observed among information criteria: (i) the AIC criterion tended to systematically choose the model with the highest number of parameters (full model); (ii) AICC (and to a certain extent, R^2^_adj_) showed moderate to good performance (frequency of success of 50% to 85%) in all the three simulation schemes, but showed also a relative bias to overselect the full model; (iii) BIC and CAIC showed a very high performance (frequency of success around 95%) in the scenario where the simplest model was right (simulation scheme 1), but often failed to select the full model when this model was right (frequency of success of only around 33%). WLD gave results similar to those of BIC and CAIC. The simulation using the maize sample showed a relative improvement in the performance of all criteria (Figure S6B). The biased preference of AIC and AICC for the full model was reduced and the frequency of success of BIC and CAIC for the full model was substantially increased with this sample (roughly 90% success). Like for pearl millet, WLD results were similar to those of BIC and CAIC.

The main difference between the pearl millet and maize samples was sample size, the maize sample being three times larger. The hypothesis concerning the impact of sample size was properly tested using inbred subsets of gradually increasing size in maize (Figure S7). We established that the frequency of success of all selection criteria (except AIC) was significantly linked to sample size (Table S7; P<10^-10^). The impact of sample size was stronger with BIC and CAIC. Note that in addition to sample size, the parameters σ_G_ and q_0_ varied with sampling. σ_G_ (involved in effect size setting) also had a significant impact on the frequency of success of all the criteria; the frequency of the background marker (q_0_) had a significant impact on selection success only for the consistent criteria (BIC, CAIC) and for R^2^_adj_ (Table S7). The impact of σ_G_ and q_0_ was lower than the impact of sample size.

**Discussion**

Model selection procedures are used to select parameters that best fit the data. The principle of parsimony is commonly associated with model selection. This principle implies limiting, as far as possible, the number of parameters in the model [S7]. Here the performance of information criteria was evaluated to select the mean structure of the mixed model. We also compared these information criteria to a procedure of model selection based on Wald test.

Our simulation highlighted the limits of information criteria when used with small samples (simulation based on pearl millet with n = 90 inbreds). In this situation, the behaviour of efficient criteria (AIC and AICC) was antagonistic to the behaviour of consistent criteria (BIC and CAIC). AIC and AICC biased the selection by wrongly preferring larger models. Conversely, BIC and CAIC tended to wrongly reject the full model. This behaviour was previously reported for a particular simulated pattern of data and seems to occur particularly when the total variance of the data is large [S3]. So in this case, efficient criteria violate parsimony while consistent criteria lead to the mistaken removal of informative parameters from the model. There is no an objective consideration that might lead to prefer one of the categories of criteria over another [S3], [S4]. Moreover it is not easy to choose which bias is more acceptable, even though certain authors consider the fact of removing relevant parameters from a model to be more serious, which implies preferring overfitting to underfitting [S3]. Methods that assess the uncertainty of model selection could help overcoming this problem [S8], [S9], [S10], but this is behind the scoop of the current study.

Nevertheless, we showed that these information criteria, notably BIC and CAIC, significantly improved their performance when used for large samples (simulation on maize, n = 277 inbreds). This is in agreement with the results of previous studies [S1], [S3], [S11]. These criteria also proved empirically as good as the procedure based on Wald test (Figure S6).

These results underlie the need of larger samples to include interaction analysis in association mapping frameworks. In a review of the last decade spanning dozens of plant species [S12], it could be seen that roughly 50% of the reported panels contained less than 100 individuals (n <100) and less than 1/5 of these panels contained more than 300 individuals (n >300).

Furthermore, this study adds empirical evidence about the adequacy of information criteria to assess fixed effects under REML. Theoretical solutions to resolve completely this issue remains addressed [S3]. In the context of the current datasets, information criteria (notably BIC and CAIC) showed a global good performance when applied on large samples. The use of these criteria could be an alternative to procedures based on Wald test, which imply multiple testing and inflate type I error.

**Supplementary references**

[S1] Hurvich CM, Tsai CL (1989) Regression and time series model selection in small samples. Biometrika, 76: 297-307.

[S2] Schwarz G (1978) Estimating the dimension of a model. The Annals of Statistics, 6: 461-464.

[S3] Gurka MJ (2006a) Selecting the best linear mixed model under REML. The American Statistician, 60(1): 19-26.

[S4] Akaike H (1974) A new look at the statistical model identification. IEEE transactions on automatic control. AC 19: 716-723.

[S5] Bozdogan H (1987) Model selection and Akaike's information criterion (AIC): the general theory and its analytical extensions. Psychometrika 52: 345-370.

[S6] Vonesh EF, Chinchilli VM, Pu K (1996) Goodness-of-fit in generalized nonlinear mixed-effects models. Biometrics 52:572-587.

[S7] Crawley MJ (2007). The R book. John Wiley & Sons, Ltd; The Atrium, Southern Gate, Chichester,West Sussex PO19 8SQ, England. 942 p.

[S8] Chatfield C (1995). Model uncertainty, data mining and statistical inference. J. R. Stat. Soc. A 158:419-466.

[S9] Posada D, Buckley TR (2004) Model selection and model averaging in phylogenetics: advantages of Akaike information criterion and Bayesian approaches over likelihood ratio tests. Systematic Biology 53 (5): 793-808.

[S10] Spiegelhalter DJ, Best NG, Carlin BP, van der Linde A (2002) Bayesian measures of model complexity and fit. J. R. Stat. Soc. B 64 (4): 583-639.

[S11] Wang J (2007) Selecting the best linear mixed model using predictive approaches. Master of Science. Brigham Young University.

[S12] Zhu C, Gore M, Buckler ES, Yu J (2008) Status and prospects of association mapping in plants. The Plant Genome 1:5-20.
